# Effects of AI-based real-time feedback on students’ motor-skill performance in physical education: a systematic review and meta-analysis

**DOI:** 10.3389/fpsyg.2026.1946151

**Published:** 2026-10-28

**Authors:** Linghui He, Lebin Li, Hanchen Wang, Zhou Wu, Jiaxing Liu, Yihao Liu

**Affiliations:** 1 College of Physical Education, Southwest University, Chongqing, China; 2 College of Teacher Education, Southwest University, Chongqing, China

**Keywords:** artificial intelligence, meta-analysis, motor-skill performance, physical education, real-time feedback, systematic review

## Abstract

**Introduction:**

Timely feedback may improve motor-skill performance in physical education, but immediate gains do not establish durable motor learning without retention or transfer assessment. This review evaluated AI-based real-time feedback for students’ post-intervention motor-skill performance in educational settings.

**Methods:**

Following PRISMA 2020, Web of Science, ERIC, PubMed, and CNKI were searched for journal articles published from 1 January 2014 to 25 June 2026. Eligible studies examined AI-based or intelligent feedback and motor-skill outcomes in educational learners using quantitative or mixed-methods designs. Risk of bias was appraised using design-specific JBI tools. Study-level Hedges’ g values were synthesized using a REML random-effects model, and certainty was assessed using GRADE.

**Results:**

Eighteen studies were included in the systematic review and 11 contributed study-level effects to the meta-analysis. One retracted report and one purely qualitative study were excluded during verification. The pooled effect favored AI-based real-time feedback (Hedges’ g = 1.484, 95% CI [0.738, 2.230]). Heterogeneity was substantial (I^2^ = 94.70%; τ^2^ = 1.50748), and the 95% prediction interval was −1.036 to 4.003. The study with the largest effect was potentially influential; excluding it reduced the pooled effect to g = 1.175 (95% CI [0.679, 1.671]). Appraisal identified recurring concerns about blinding, allocation concealment, follow-up, and statistical reporting.

**Discussion:**

AI-based real-time feedback showed a positive average association with post-intervention motor-skill performance, but extreme heterogeneity and a prediction interval crossing zero limit conclusions about effects in individual settings. None of the pooled outcomes assessed delayed retention or transfer, so durable motor learning cannot be inferred. GRADE certainty was very low due to serious risk of bias, very serious inconsistency, and serious indirectness. The review is registered in PROSPERO (CRD420261477139).

**Systematic review registration:**

https://www.crd.york.ac.uk/PROSPERO/view/CRD420261477139, PROSPERO CRD420261477139.

## 1 Introduction

Motor-skill performance is an immediate and observable outcome of physical-education instruction, reflecting how effectively a learner executes a movement under particular task and assessment conditions. Feedback is one of the main means through which students detect movement errors and adjust subsequent attempts during practice (Newell, 1991; Salmoni et al., 1984; Wulf et al., 2010). Short-term performance change, however, should not be equated with motor learning. In motor-learning research, learning is inferred from changes that persist beyond the immediate practice context and is therefore more appropriately examined through delayed retention and/or transfer assessments (Salmoni et al., 1984; Winstein and Schmidt, 1990). This distinction is central to the present review because the outcomes contributing to the primary meta-analysis were post-intervention motor-skill performance measures rather than delayed retention or transfer outcomes. Within this more limited outcome domain, feedback can guide attention, calibrate error detection, and help learners modify movement during subsequent practice attempts (Black and Wiliam, 1998; Hattie and Timperley, 2007; Kluger and DeNisi, 1996; Shute, 2008). In large or heterogeneous classes, however, teachers must observe many learners at once, identify movement errors quickly, and decide what information is useful at the moment of practice. This makes precise, timely, and individualized feedback difficult to provide consistently.

AI-based technologies have begun to enter this instructional space as part of broader developments in artificial intelligence in education (Chen et al., 2020a; Hwang et al., 2020; Zawacki-Richter et al., 2019). Computer vision, pose estimation, motion recognition, deep learning, wearable sensors, and intelligent tutoring or assessment systems can capture aspects of movement, compare them with criteria or learned patterns, and return visual, verbal, dashboard, or corrective information (Cao et al., 2017; Colyer et al., 2018; Mathis et al., 2018; Roll and Wylie, 2016). These systems do not make feedback automatically pedagogically meaningful. Their possible contribution is more specific: they may shorten the interval between movement execution and correction, make subtle movement deviations visible, and support more consistent monitoring across repeated practice trials (Sigrist et al., 2013; van der Kruk and Reijne, 2018).

The current evidence base is dispersed across educational levels, sport domains, AI technologies, feedback modalities, and research designs. Some studies evaluate controlled teaching interventions, whereas others focus on intelligent systems, smart classrooms, or applied teaching models with limited extractable quantitative data. Existing reviews of AI in education and movement-analysis technologies help map broader trends, but they do not directly estimate whether AI-based real-time feedback improves students’ post-intervention motor-skill performance in physical education (Chen et al., 2020b; Hwang et al., 2020; Zawacki-Richter et al., 2019).

The review addressed four research questions:

RQ1: What is the overall effect of AI-based real-time feedback on students’ post-intervention motor-skill performance in physical education?
RQ2: What AI technologies and feedback modalities have been used to support motor-skill outcomes?
RQ3: Do the effects differ across motor skill outcome categories, educational levels, or feedback types?
RQ4: What are the methodological characteristics and limitations of the current evidence base?

## 2 Literature review

### 2.1 Motor-skill performance, motor learning, and feedback in physical education

Motor-skill performance and motor learning are related but conceptually distinct. Motor-skill performance refers to the observable execution of a skill at a particular point in time and under particular task or assessment conditions. Motor learning, by contrast, concerns changes that persist beyond the immediate practice context and is conventionally inferred from delayed retention and/or transfer rather than from post-practice performance alone (Salmoni et al., 1984; Winstein and Schmidt, 1990). Short-term improvement can occur during the learning process, but it does not by itself show that the change has been retained or generalized. Accordingly, this review classifies immediate and post-intervention measures as motor-skill performance outcomes and reserves the term motor learning for outcomes supported by delayed retention and/or transfer assessments.

Augmented feedback can affect immediate performance and may also contribute to longer-term learning, but these outcomes are not interchangeable. Feedback provided during or soon after movement can help learners detect errors, modify a subsequent attempt, and improve performance within the instructional episode. Whether that improvement persists after feedback is removed or transfers to a new task is a separate empirical question. Feedback timing, frequency, attentional focus, and task complexity may influence practice performance and longer-term learning differently; more frequent or more immediate feedback is therefore not necessarily synonymous with better learning (Salmoni et al., 1984; Winstein and Schmidt, 1990; Wulf and Prinz, 2001; Wulf and Shea, 2002).

Traditional feedback in physical education has practical limitations. It may be delayed, subjective, unevenly distributed across students, or too general to guide a specific movement correction. Teachers may prioritize the most visible errors or the students who ask for help, while quieter or less skilled learners receive less individualized information. Video replay can improve visibility, but without structured analysis it still requires the teacher or student to decide what matters. These constraints motivate interest in systems that can detect movement features and return feedback during or immediately after practice.

### 2.2 AI-based technologies in physical education

AI-based technologies in education are used to analyze, interpret, or evaluate learner-related information in support of teaching, assessment, and feedback (Hwang et al., 2020). Relevant approaches include computer vision, pose or motion recognition, machine-learning-based analysis, intelligent teaching or assessment systems, and generative or multimodal AI. These technologies build on wider developments in intelligent tutoring, automated assessment, and learning analytics, but their use in physical education depends on whether bodily movement can be captured, interpreted, and translated into usable feedback (Kulik and Fletcher, 2016; VanLehn, 2011).

For motor-skill instruction, AI-supported systems have been used to identify posture or movement deviations through camera-based pose estimation and visual recognition (Hsia et al., 2024, 2025; Lu et al., 2025; Ma et al., 2025a). An IMU-based Baduanjin assessment system has also used wearable motion capture and machine-learning models to recognize movement patterns and evaluate execution accuracy (Ma et al., 2025b). Other applications have provided automated performance assessment, visual feedback, or mobile app-based feedback during physical education and motor-skill practice (Huang et al., 2026; Lin et al., 2025; Ma et al., 2025a).

The technical basis for these systems varies. Pose-estimation and markerless movement-analysis methods can identify body landmarks or movement patterns from video, whereas wearable inertial sensors can provide information about movement timing, acceleration, orientation, or execution outside a laboratory setting (Camomilla et al., 2018; Cao et al., 2017; Iosa et al., 2016; Mathis et al., 2018). Sports and movement-analysis reviews show both the promise and the measurement constraints of these tools, including accuracy, calibration, context sensitivity, and the difficulty of translating raw movement data into pedagogically meaningful cues (Colyer et al., 2018; van der Kruk and Reijne, 2018).

### 2.3 Real-time feedback and motor-skill performance

Consistent with augmented-feedback literature, feedback timing was conceptualized in instructional rather than engineering terms (Sigrist et al., 2013). In this review, real-time or immediate feedback included augmented feedback delivered concurrently with movement performance or sufficiently soon after an attempt to inform ongoing practice or a subsequent corrective attempt within the same instructional cycle. Because such feedback operates in close temporal proximity to performance, its most directly observable outcome is change in ongoing or post-intervention motor-skill performance. Whether that change persists as motor learning cannot be determined without delayed retention and/or transfer assessments.

Exact latency was not required when the full text showed that feedback remained integrated with the instructional or practice cycle. End-of-course retrospective reports, purely summative analytics, and researcher-only offline analyses not returned to learners or teachers were outside this construct. Real-time feedback may be instructionally useful because it can make movement errors visible and provide information for the next attempt, but greater immediacy or frequency should not automatically be interpreted as producing stronger learning. Motor-learning research indicates that augmented feedback can improve practice performance while frequent feedback may not produce corresponding advantages in retention or transfer (Salmoni et al., 1984; Winstein and Schmidt, 1990).

A meta-analysis is needed because individual studies vary in sport domain, intervention duration, education level, technology type, feedback timing, and outcome measurement. A narrative synthesis can describe these differences, but it cannot estimate the overall magnitude of the association between AI-based real-time feedback and motor skill outcomes. At the same time, a pooled estimate must be interpreted alongside heterogeneity, because a single average effect may hide meaningful variation across contexts.

### 2.4 Need for the present systematic review and meta-analysis

Existing reviews of AI in education, intelligent tutoring, and digital movement analysis help clarify the technological landscape, but they do not directly answer whether AI-based real-time feedback improves students’ post-intervention motor-skill performance in physical education (Chen et al., 2020a; Chen et al., 2020b; Hwang et al., 2020; Kulik and Fletcher, 2016). The specific intersection of AI-based movement analysis, temporally proximal feedback, and students’ motor-skill performance therefore requires a narrower synthesis. This focus is deliberately narrower than motor learning: the available intervention literature primarily reports immediate or post-intervention performance, whereas stronger claims about durable learning require retention and/or transfer evidence.

This review therefore separates the empirical question of post-intervention performance from the broader question of motor learning. It examines whether AI-based real-time feedback is associated with better motor-skill performance after intervention, what types of AI and feedback have been used, and how strong the current evidence base is. Whether any observed performance advantage persists as durable motor learning is treated as a distinct question that cannot be answered by the primary meta-analytic outcomes.

## 3 Methods

### 3.1 Review design and reporting framework

This study was designed as a systematic review and meta-analysis of empirical evidence on AI-based real-time feedback and students’ motor-skill outcomes in physical education. The review followed the PRISMA 2020 framework for search reporting, screening, eligibility assessment, and synthesis (Page et al., 2021). The protocol was implemented as a reproducible screening, extraction, and synthesis workflow, with explicit tracking of non-journal exclusions, full-text exclusions, and the post-verification removal of one retracted article. The review combined a narrative synthesis of all included studies with a quantitative meta-analysis of studies that provided sufficient data to estimate a motor skill effect size. The review protocol is registered in PROSPERO (CRD420261477139).

### 3.2 Search strategy

Searches were conducted in Web of Science, ERIC, PubMed, and CNKI. The search window covered January 1, 2014 to June 25, 2026. The lower publication-date boundary was defined when the search strategy was initially developed, before study screening, to delimit the contemporary technology-focused scope of the review. Specifically, the review addressed AI-based educational feedback systems that integrate data-driven movement analysis, automated performance assessment, and corrective information relevant to motor-skill learning, rather than attempting a historical synthesis of all computer-assisted feedback approaches. The 2014 starting point also corresponds to a period of important developments in learning-based human pose estimation; for example, DeepPose demonstrated a deep-neural-network approach to predicting body-joint locations (Toshev and Szegedy, 2014). This paper is cited as contextual support for the technological focus, not as an assertion that AI-based or automated feedback was unavailable before 2014. The same publication-date restriction was applied across all four databases. English-language database searches focused on journal articles and used terms related to artificial intelligence, machine learning, deep learning, computer vision, pose estimation, motion capture, wearable sensors, intelligent systems, feedback, physical education, motor skills, motor learning, movement skills, and students or learners. CNKI searches used Chinese terms for artificial intelligence, smart physical education, posture recognition, movement recognition, feedback, correction, evaluation, physical education, sport skill learning, and students.

The review included English and Chinese journal articles. For CNKI, only Chinese academic journal articles were retained. Theses, dissertations, conference papers, books, patents, standards, and newspapers were excluded before screening to maintain consistency with the journal-article eligibility criteria. The complete search strategy is reported in Supplementary Table S1.

### 3.3 Eligibility criteria

Eligibility criteria were defined using the PICOS framework. The population was students or learners in physical education or educational motor-skill contexts. For the purposes of this review, and drawing on AI-in-education literature (Hwang et al., 2020), ‘AI-based’ referred to educational feedback interventions in which AI or intelligent computational technologies were used to analyze, interpret, evaluate, or otherwise transform learner- or movement-related information in support of feedback, assessment, or motor-skill correction. This included computer vision, pose or motion recognition, machine-learning-based analysis, intelligent teaching or assessment systems, generative or multimodal AI, and sensor-, wearable-, or simulation-based systems when an intelligent analytic or feedback function formed part of the intervention. Cameras, wearables, sensors, dashboards, virtual-reality environments, smart equipment, and digital platforms were not considered AI-based solely by virtue of the device itself. Consistent with augmented-feedback literature (Sigrist et al., 2013), ‘real-time or immediate feedback’ referred to augmented feedback delivered concurrently with movement performance or in sufficiently close temporal proximity after an attempt within the same instructional or practice cycle, such that it could support ongoing practice or subsequent motor correction. Feedback could be system-generated or teacher- or peer-mediated when AI-derived information materially informed the feedback. Eligible comparisons included traditional physical education instruction, usual teaching, teacher-led feedback, non-AI feedback, conventional practice, or other comparison conditions. Eligible outcomes were motor-skill outcomes, including movement quality, technique execution, sport-specific skill performance, fundamental motor skills, action accuracy, skill proficiency, or motor performance. Eligible study designs were original empirical quantitative or mixed-methods studies with relevant motor-skill data. Immediate or post-intervention assessments were classified as motor-skill performance outcomes. Outcomes were classified as evidence of motor learning only when they involved delayed retention and/or transfer assessments. Where studies reported both immediate performance and retention or transfer outcomes, these outcomes were extracted and classified separately.

Studies were excluded if they focused on medical, rehabilitation, surgical training, clinical patients, elite athlete training outside an educational context, theoretical discussion, reviews, pathway or model discussions without empirical testing, pure platform design without student motor-skill outcomes, or studies without explicit motor-skill outcomes. Studies with insufficient quantitative information for effect-size calculation could still be retained for the systematic review if they met the broader empirical and topical eligibility criteria.

### 3.4 Study selection

Records were imported from the four databases, deduplicated, and screened by title and abstract against the eligibility criteria. Full texts were then assessed for potentially eligible records. The database search identified 1,009 records. Before screening, 82 duplicate records and 100 CNKI non-journal records were removed. A total of 827 records were screened by title and abstract, of which 725 were excluded. Full texts were sought for 102 reports; six were unavailable. Ninety-six full-text reports were assessed for eligibility, and 78 were excluded with reasons, including one retracted article identified during reference verification, one purely qualitative participatory-action-research study excluded during revision eligibility verification, and two CNKI reports excluded after independent eligibility reassessment (details in Supplementary Table S2) because the full-text reports did not sufficiently establish an implemented AI-based feedback intervention with verifiable student motor-skill outcomes. The final synthesis included 18 studies, of which 11 contributed study-level effect sizes to the meta-analysis. The selection process is shown in Figure 1, and full-text exclusion reasons are summarized in Supplementary Table S2. Title and abstract screening of all 827 records and full-text eligibility assessment of all 96 retrieved reports were independently performed by Linghui He and Lebin Li. At both screening stages, any differences in eligibility judgments were resolved through discussion and consensus before the final study set was established.

**Figure 1:**
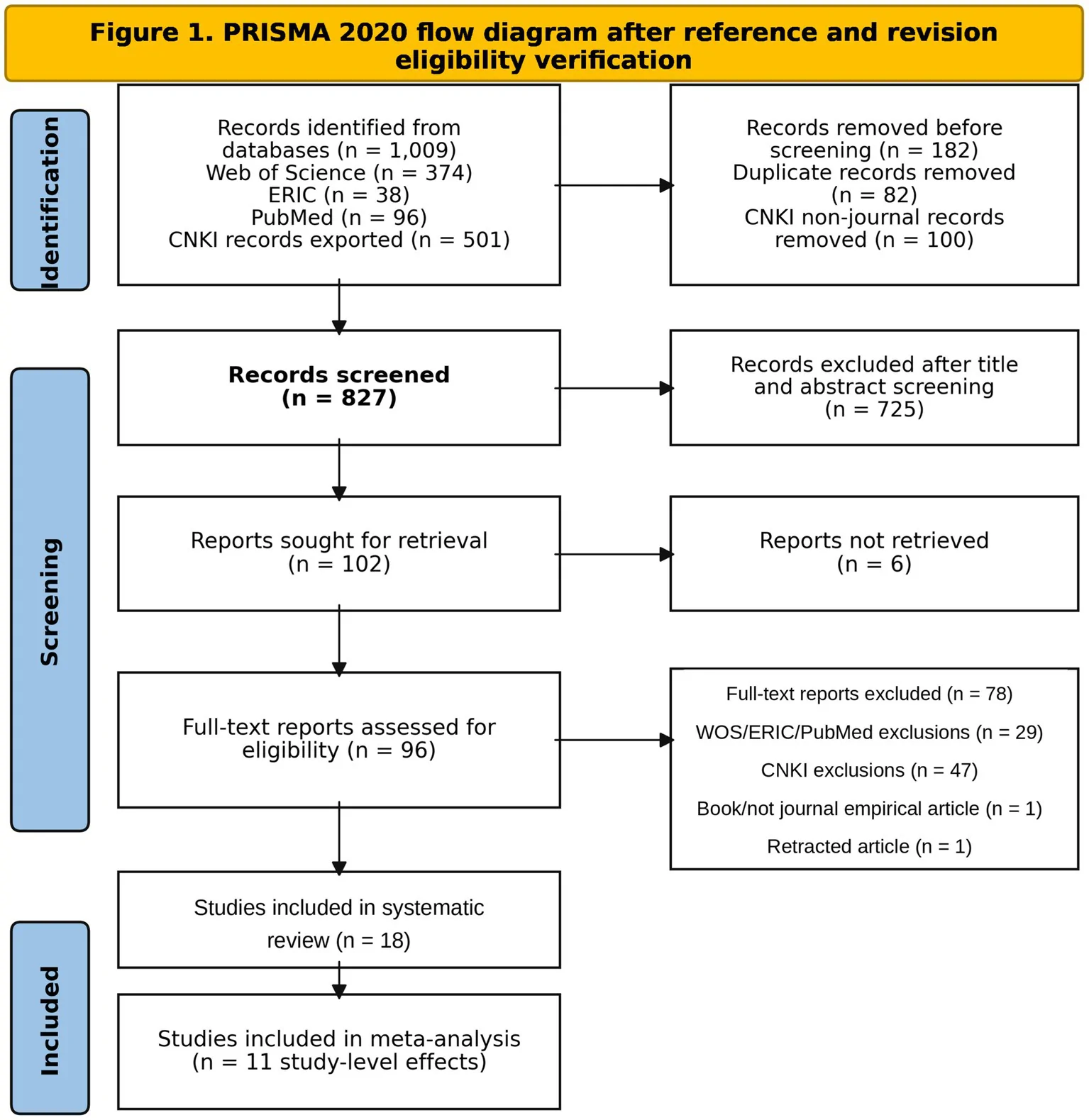
PRISMA 2020 flow diagram after reference and revision eligibility verification (Page et al., 2021).

### 3.5 Data extraction

For all 18 retained studies, data were extracted on study identity, year, country or region, education level, participants, sample size, study design, physical education or sport context, AI technology, feedback timing and modality, intervention duration, comparison condition, motor skill outcomes, and inclusion status. For studies eligible for meta-analysis, group-level data or convertible statistics were extracted for motor skill outcomes. The extraction file also retained page or table sources and notes on calculation or conversion where available. During this revision, outcome assessment timing was additionally verified against the full texts; each reported motor-skill outcome was classified as immediate/during-practice performance, post-intervention performance, retention, transfer, or unclear. Studies without an assessable motor-skill outcome were marked not applicable. During this revision, intervention descriptions were re-checked against the full texts to verify the AI or intelligent function, feedback modality, feedback recipient, temporal relation to practice, and teacher or peer mediation. Data extraction for the 18 retained studies was completed by Linghui He and Lebin Li and systematically cross-checked against the original study reports and finalized extraction files. Discrepant or unclear entries were rechecked against the source reports and resolved through discussion and consensus before synthesis.

The meta-analysis used one study-level effect size per study. Lin et al. (2025) reported multiple dependent motor-skill outcomes, so these outcomes were first synthesized within study before entry into the main model. The primary within-study synthesis assumed a within-study outcome correlation of *r* = 0.50; sensitivity analyses examined *r* = 0.30 and *r* = 0.70. Sun and Lin (2026) was retained for narrative synthesis only because reliable information required to compute Hedges’ g and its sampling variance was unavailable. Two CNKI reports were excluded from the systematic review and meta-analysis after independent eligibility reassessment of their full-text reports (Supplementary Table S2). Their reported intervention implementation and student motor-skill outcome data could not be sufficiently verified against the prespecified eligibility criteria. One retracted article was excluded from both the systematic review and meta-analysis after reference verification. The outcome-level data, effect-size calculation inputs, and selected study-level effects included in the meta-analysis are detailed in Supplementary Table S3.

### 3.6 Risk-of-Bias and methodological appraisal

Risk of bias and methodological limitations were reassessed using JBI tools matched to the underlying design of each retained study (Aromataris et al., 2024). Randomized controlled trials, including class-level randomized trials, were appraised using the revised JBI critical appraisal tool for randomized controlled trials (Barker et al., 2023); quasi-experimental and non-randomized intervention studies were appraised using the revised JBI critical appraisal tool for quasi-experimental studies (Barker et al., 2024); and the analytical cross-sectional/system-validation study was appraised using the revised JBI analytical cross-sectional tool (Barker et al., 2026). Study design classification was verified from the full-text methods rather than relying solely on the design labels used by the original authors. Each item was judged as Yes, No, Unclear, or Not applicable at the relevant study, outcome, or result level. Percentage scores and global high/moderate/low quality categories were not calculated; instead, item- and domain-level judgments were retained to preserve design-specific sources of bias and reporting uncertainty. Detailed design-specific judgments are reported in Supplementary Table S4. Risk-of-bias assessment was independently performed by Linghui He and Lebin Li. Item-level judgments were compared, and any differences were resolved through discussion and consensus.

### 3.7 Certainty of evidence (GRADE)

Certainty of the evidence for the primary meta-analytic outcome, post-intervention motor-skill performance, was assessed using the Grading of Recommendations Assessment, Development and Evaluation (GRADE) framework (Guyatt et al., 2011; Balshem et al., 2011). The assessment considered risk of bias, inconsistency, indirectness, imprecision, and publication bias at the outcome level. Because the primary pooled model combined nine studies classified as randomized trials and two quasi-experimental studies, the certainty assessment was anchored to the randomized evidence and informed by a randomized-trial-only sensitivity analysis to determine whether inclusion of the two quasi-experimental studies materially altered the inference. Downgrading decisions were documented explicitly, and the final certainty was expressed as high, moderate, low, or very low. GRADE certainty was treated as a body-of-evidence judgment and was kept conceptually distinct from the design-specific JBI appraisal of individual studies. Detailed GRADE judgments are provided in Supplementary Table S5.

### 3.8 Effect size calculation and statistical analysis

The effect size was Hedges’ g, a small-sample-corrected standardized mean difference (Hedges, 1981; Borenstein et al., 2009). For outcomes with complete experimental and control post-test means, standard deviations, and sample sizes, standardized mean differences were computed using the pooled post-test standard deviation and corrected for small-sample bias. Sampling variance was calculated for each Hedges’ g. Where lower scores represented better performance, effect directions were reversed so that positive values consistently favored AI-based real-time feedback. Accordingly, the primary meta-analytic estimand was the average between-group standardized effect of a functionally defined but technologically heterogeneous class of AI-supported timely feedback interventions on post-intervention motor-skill performance. The interventions were grouped on the basis of their shared instructional feedback function rather than technological equivalence. Accordingly, the pooled estimate should not be interpreted as the effect of any single AI technology or feedback modality.

For studies with multiple dependent motor-skill outcomes, result-level effects were retained for transparency but were not entered as independent studies in the primary random-effects model. Instead, effects were combined within study before pooling. The primary model used a REML random-effects estimator, rather than treating all studies as estimating a single common effect. Conceptual grouping was based on the shared instructional feedback function described above; the random-effects model was then used to accommodate expected variation within that class across technological implementations, populations, motor tasks, and feedback formats. Random-effects interpretation followed standard guidance for heterogeneous evidence syntheses (Borenstein et al., 2009; Borenstein et al., 2010; DerSimonian and Laird, 1986; Viechtbauer, 2010). Heterogeneity was assessed using Q, I^2^, τ^2^, τ, and prediction intervals because I^2^ alone does not describe the expected dispersion of true effects across contexts (Higgins and Thompson, 2002; Higgins et al., 2003; IntHout et al., 2016). Sensitivity analyses included leave-one-out models, within-study correlation assumptions for Lin et al. (2025), and influence diagnostics using studentized residuals, Cook’s distance, and hat values. Funnel plot inspection, Egger’s regression test, and Begg’s rank-correlation test were treated as exploratory because only 11 studies were included in the main model and small-study-effect tests are unstable with small samples (Begg and Mazumdar, 1994; Egger et al., 1997; Sterne et al., 2011).

## 4 Results

### 4.1 Study selection and characteristics

During reference verification, one retracted article was identified and excluded from the final synthesis. Two previously included CNKI reports (listed in Supplementary Table S2) were also excluded after two-reviewer full-text eligibility reassessment because their AI-feedback implementation and student motor-skill results could not be sufficiently verified. The revised PRISMA process yielded 18 studies for the systematic review and 11 studies for the meta-analysis (Figure 1). The final corpus comprised English- and Chinese-language reports identified through Web of Science, ERIC, PubMed, and CNKI.

The included educational and sport-specific settings comprised yoga or yogism (Hsia et al., 2024; Hsia et al., 2025; Huang et al., 2026), badminton (Hsu et al., 2025; Lin et al., 2025), dance (Xu et al., 2025), cheerleading (Lu et al., 2025), Baduanjin (Ma et al., 2025a; Ma et al., 2025b), and handball (Rakha, 2026). Ball-sport settings included soccer or football (Bouzid et al., 2026; Sun and Lin, 2026; Zhong, 2026), basketball (Lin, 2026; Sun and Lin, 2026), tennis (Sun and Lin, 2026), and table tennis (Lin, 2024). Additional contexts included primary-school fundamental motor skills (Li, 2025), multisport training involving swimming and shot put (Shen, 2026), and broader smart physical education or assessment systems (Zhang et al., 2025). Study characteristics are summarized in Table 1 and Supplementary Table A1.

**Table 1:** Summary characteristics of included studies.

Characteristic	Category	Number of studies	Notes
Countries or regions	Mainland China (11); Taiwan (5); Saudi Arabia (1); Tunisia and international youth sport context (1)	18	Counts were recalculated from the 18 retained study-level records in Supplementary Table A1.
Education level	University and college; vocational; primary, middle, and high school; youth sport or mixed settings	University and college 13; other or mixed 5	Most studies were university PE or college sport-skill contexts; school and vocational settings were less frequent.
Study design	Controlled, quasi-experimental, randomized, or comparative designs; system-development or implementation studies	Controlled or comparative 15; descriptive or development-oriented 3	The main meta-analysis retained only studies with extractable study-level motor-skill effects.
Sport or motor skill domains	Yoga and yogism, Baduanjin, badminton, cheerleading, soccer and football, handball, basketball, table tennis, dance, and fundamental motor skills	18	Domains were diverse, contributing to conceptual and statistical heterogeneity.
AI technology categories	Computer vision and pose or motion recognition; intelligent teaching or feedback systems; wearable and sensor-based systems; virtual simulation and VR; generative AI or smart platforms	18	Several interventions combined more than one technology layer, so categories are descriptive rather than mutually exclusive.
Feedback modalities	Real-time or immediate visual, video, and dashboard feedback; automated corrective feedback; wearable or sensor feedback; text, verbal, or mixed feedback	Immediate and real-time variants 16; mixed and process variants 3	Visual, video, dashboard, score, skeleton, sensor, and multimodal feedback were common. Feedback-timing categories are not mutually exclusive; mixed or process-oriented systems may also provide immediate feedback.
Outcome categories	Movement quality and technique execution (7); Sport-specific skill performance (4); Other (6); Fundamental motor skills (1)	18	Movement quality and technique execution was the most common outcome category.
Inclusion type	Meta-analysis; systematic-review-only narrative synthesis	Meta-analysis 11; systematic review only 7	Seven studies were retained for narrative synthesis only; two CNKI reports were excluded after independent eligibility reassessment (Supplementary Table S2), in addition to one previously excluded qualitative study and one retracted article.

Detailed study-level characteristics are provided in Supplementary Table A1.

Most included studies were conducted in university settings (13/18), with smaller numbers from primary, secondary, high-school, vocational, youth sport, or mixed educational contexts. The evidence base was concentrated in Chinese and East Asian educational settings, with additional studies from Saudi Arabia, Tunisia, or international youth sport contexts. Eleven studies were included in the meta-analysis, and 7 were retained for systematic review only.

### 4.2 Overall effect of AI-based real-time feedback on motor-skill performance

The primary random-effects meta-analysis included 11 study-level effect sizes after removal of the retracted article. The model yielded a positive average pooled effect for students’ post-intervention motor-skill performance (Hedges’ g = 1.484, 95% CI [0.738, 2.230], *p* < 0.001). The confidence interval remained entirely above zero, supporting a positive average effect across the included studies. This inference concerns the average effect and should be distinguished from the dispersion of true effects across settings. All pooled study-level effects represented post-intervention motor-skill performance; neither retention nor transfer outcomes entered the primary model.

The included studies differed in design and intervention implementation, and statistical heterogeneity was high (Q = 188.565, df = 10, *p* < 0.001; I^2^ = 94.70%; τ^2^ = 1.50748; τ = 1.228). The 95% prediction interval was −1.036 to 4.003. Unlike the confidence interval, which quantifies uncertainty around the average pooled effect, the prediction interval reflects the range within which the true effect of a new study conducted under conditions comparable to those represented in the meta-analysis may plausibly fall (IntHout et al., 2016). Because the prediction interval crossed the null and spanned effects from potentially unfavorable to very large positive values, it indicates substantial variation in true effects across settings. The pooled estimate should therefore be interpreted as a positive average across highly heterogeneous true effects rather than as a stable or uniformly expected intervention effect in every physical-education context. The study-level effects and pooled estimate are shown in Figure 2 and Table 2.

**Figure 2:**
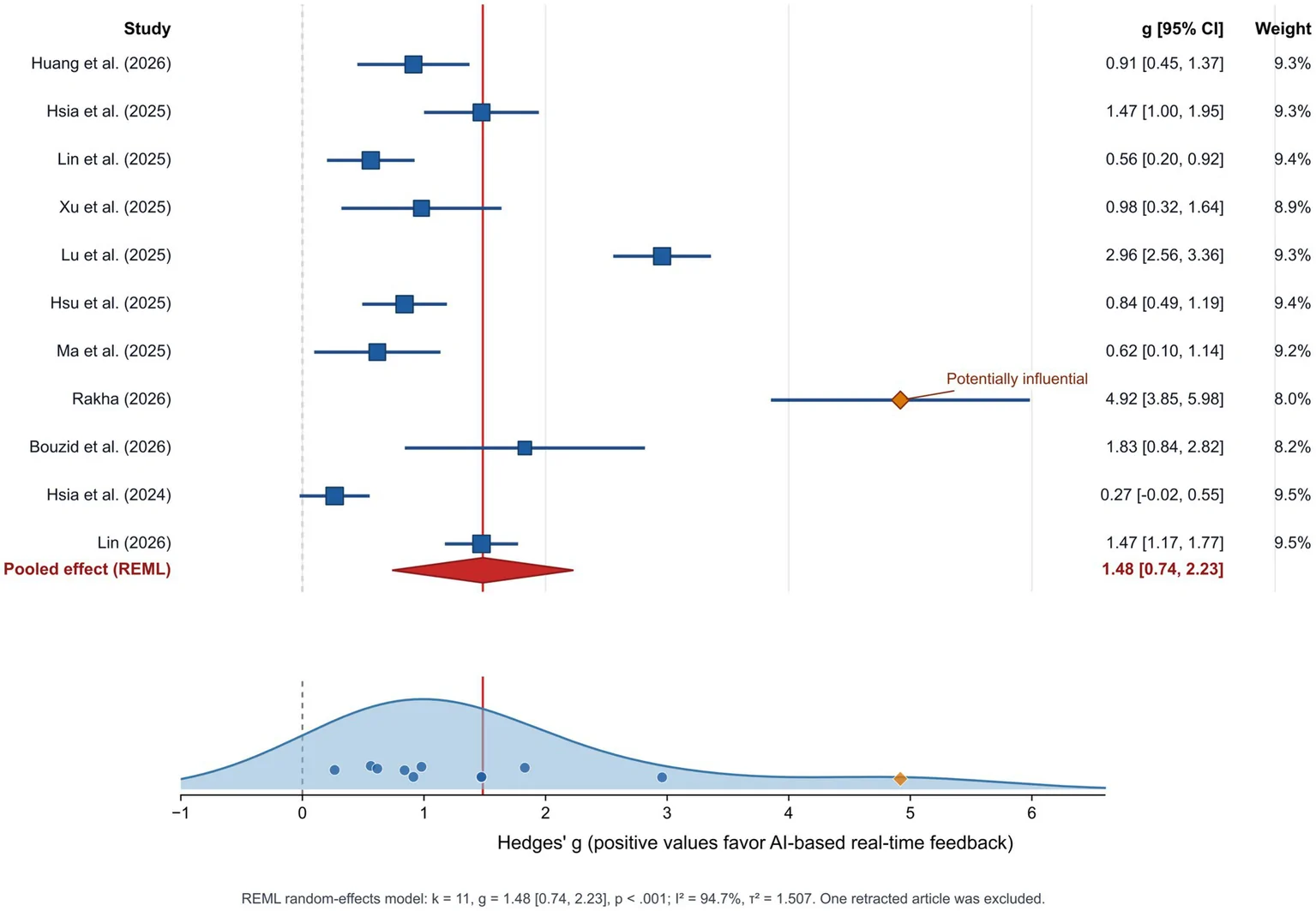
Raincloud forest plot of AI-based real-time feedback on post-intervention motor-skill performance. Squares represent study-level Hedges’ *g* values, horizontal lines represent 95% confidence intervals, and point sizes reflect random-effects weights. The red diamond indicates the pooled REML random-effects estimate. Positive values favor AI-based real-time feedback. Rakha (2026) was flagged as a potentially influential study in influence diagnostics.

**Table 2:** Study-level effect sizes included in the meta-analysis.

Study	Hedges’ g	95% CI	Weight %	Outcome category	Notes
Bouzid et al. (2026)	1.830	0.842 to 2.817	8.2	Movement quality and technique execution	Positive values favor AI-based real-time feedback.
Huang et al. (2026)	0.913	0.452 to 1.375	9.3	Movement quality and technique execution	Positive values favor AI-based real-time feedback.
Lin (2026)	1.473	1.172 to 1.774	9.5	Sport-specific skill performance	Positive values favor AI-based real-time feedback.
Rakha (2026)	4.918	3.853 to 5.984	8.0	Sport-specific skill performance	Potentially influential in diagnostics; retained in the main model.
Hsia et al. (2025)	1.473	1.001 to 1.946	9.3	Movement quality and technique execution	Positive values favor AI-based real-time feedback.
Hsu et al. (2025)	0.840	0.492 to 1.189	9.4	Sport-specific skill performance	Positive values favor AI-based real-time feedback.
Lin et al. (2025)	0.562	0.202 to 0.923	9.4	Sport-specific skill performance	Study-level synthesis of two badminton outcomes before pooling.
Lu et al. (2025)	2.959	2.556 to 3.361	9.3	Sport-specific skill performance	Positive values favor AI-based real-time feedback.
Ma et al. (2025a)	0.616	0.097 to 1.136	9.2	Other	Positive values favor AI-based real-time feedback.
Xu et al. (2025)	0.979	0.321 to 1.638	8.9	Movement quality and technique execution	Positive values favor AI-based real-time feedback.
Hsia et al. (2024)	0.265	−0.023 to 0.553	9.5	Movement quality and technique execution	Positive values favor AI-based real-time feedback.

The revised main model used 11 study-level Hedges’ g estimates. One retracted article was removed before analysis; Sun and Lin (2026) was retained for narrative synthesis only; two CNKI reports were excluded after eligibility reassessment (Supplementary Table S2).

### 4.3 AI technologies and feedback modalities in the included studies

The included studies employed diverse AI or intelligent systems (Supplementary Table A2). Computer vision, pose estimation, or movement recognition supported assessment and feedback in yoga or yogism (Hsia et al., 2024; Hsia et al., 2025; Huang et al., 2026), badminton (Hsu et al., 2025; Lin et al., 2025), dance (Xu et al., 2025), cheerleading (Lu et al., 2025), and Baduanjin (Ma et al., 2025a). Three-dimensional motion capture also informed soccer-tackling feedback (Bouzid et al., 2026). Wearable inertial sensors were used for Baduanjin formative assessment (Ma et al., 2025b), while smart equipment and wearable-enabled platforms featured in fundamental-motor-skill and table-tennis settings (Li, 2025; Lin, 2024). Virtual simulation and motion-analysis systems appeared in mixed-sport and football instruction (Sun and Lin, 2026; Zhong, 2026). Smart classrooms, platform-based assessment, wearable/augmented-reality technologies, and other AI-supported instructional systems were described in further physical education contexts (Lin, 2026; Shen, 2026; Zhang et al., 2025). Generative or multimodal AI approaches were reported in university physical education and handball teaching (Rakha, 2026).

Feedback modalities also varied by study (Supplementary Table A2). Yoga systems displayed skeleton visualizations, radar charts, scores, or rankings (Hsia et al., 2024; Hsia et al., 2025), while gamified yogism and Baduanjin interventions used immediate visual feedback, dashboards, or automated performance scores (Huang et al., 2026; Ma et al., 2025a). Badminton studies used mobile-app and video-based feedback (Hsu et al., 2025; Lin et al., 2025); dance and soccer-tackling studies used visual or video feedback (Xu et al., 2025; Bouzid et al., 2026). Visual, auditory, haptic, or vibration-based feedback was described in cheerleading and broader sport-training systems (Lu et al., 2025; Shen, 2026). Sensor- or dashboard-mediated information was incorporated into IMU-based assessment and smart teaching environments (Ma et al., 2025b; Lin, 2026; Sun and Lin, 2026; Zhong, 2026). In handball teaching, ChatGPT-generated feedback was mediated by peers during reciprocal instruction (Rakha, 2026).

Across the 11 meta-analyzed studies, computer vision or pose/motion recognition accounted for nine study-level effects, while intelligent feedback or teaching systems accounted for two (Table 2; Supplementary Table A2). The principal feedback categories in the quantitative synthesis were visual/video feedback and real-time/immediate feedback. Although technological implementations differed, the interventions were classified under a common higher-order feedback construct only when an AI or intelligent function analyzed or interpreted learner- or movement-related information and informed feedback delivered within the instructional or practice cycle. These technology categories were descriptive and did not imply that the interventions were technically equivalent. All 11 studies remained in the primary meta-analysis.

### 4.4 Effects across motor skill outcomes and educational contexts

Outcome categories varied across the 18 retained studies. Movement quality or technique execution outcomes appeared in 7 studies, sport-specific skill performance in 4, fundamental motor skills in 1, and other motor-skill outcomes in 6. In the exploratory meta-analytic subgroup results, movement quality or technique execution outcomes and sport-specific skill performance outcomes both showed positive pooled estimates. These subgroup estimates should be interpreted descriptively because the number of studies in each subgroup was small and heterogeneity remained high.

Exploratory subgroup analyses were conducted only when at least three studies were available. Computer vision or pose and motion recognition, visual and video feedback, real-time and immediate feedback, university-based studies, movement-quality outcomes, and sport-specific skill outcomes each showed positive pooled estimates. The intelligent feedback or teaching system subgroup contained only two studies after removal of the retracted article and was therefore described rather than formally pooled.

Positive pooled estimates were observed across several sport, technology, and feedback categories; however, the small number of studies within individual subgroups and the substantial residual heterogeneity preclude conclusions regarding the consistency or generalizability of effects across sports or feedback formats. These subgroup findings should therefore be considered exploratory and hypothesis-generating rather than evidence of definitive moderators.

### 4.5 Risk of Bias and methodological appraisal of the evidence base

Design-specific appraisal results are summarized in Table 3 and Supplementary Table S4. Across the 18 retained studies, 201 design-specific JBI items were appraised: 141 were judged Yes, 34 Unclear, 23 No, and 3 Not applicable. These are item-level judgments and were not converted into study-level percentage scores or global high/moderate/low quality categories. Among the 10 randomized controlled trials, participant and intervention-provider blinding were not achieved in all 10 studies, allocation concealment was unclear in 9, and outcome-assessor blinding was unclear in 7. Among the 7 quasi-experimental or non-randomized intervention studies, follow-up completeness was unclear in 3, outcome-measurement reliability was unclear in 2, and statistical analysis was judged No in 2; one study lacked a control group. The single analytical cross-sectional/system-validation study met all eight applicable items as reported. These findings are reported as design-specific methodological judgments rather than as a single study-quality score.

**Table 3:** Design-specific risk-of-bias and methodological appraisal summary.

Design group	Studies (meta / narrative)	Appraisal tool	Key item-level findings	Interpretation
Randomized controlled trials	10 (9/1)	Revised JBI RCT tool	Participant blinding: No 10/10; intervention-provider blinding: No 10/10; allocation concealment: Unclear 9/10; outcome-assessor blinding: Unclear 7/10.	Important risks or reporting uncertainties remained despite otherwise structured trial designs.
Quasi-experimental / non-randomized intervention studies	7 (2/5)	Revised JBI quasi-experimental tool	Follow-up: Unclear 3/7; outcome reliability: Unclear 2/7; statistical analysis: No 2/7; control group absent in 1/7.	Methodological limitations were more heterogeneous and were concentrated in several narrative-only studies.
Analytical cross-sectional / system-validation study	1 (0/1)	Revised JBI analytical cross-sectional tool	Eight of eight applicable items were judged Yes.	Retained as narrative technology/assessment evidence rather than causal intervention-effect evidence.

Appraisal tools were matched to study design. No percentage-based overall score or global high/moderate/low quality classification was used.

Methodological limitations were not confined to narrative-only studies. Within the meta-analysis subset, recurrent concerns related mainly to blinding and allocation concealment, while several narrative-only studies had additional uncertainty regarding control structures, participant flow, outcome-measurement reliability, and statistical analysis. These judgments were therefore retained at the item and domain level rather than compressed into a global quality category.

### 4.6 Certainty of evidence (GRADE)

GRADE certainty was assessed for the pooled post-intervention motor-skill performance outcome. The evidence was rated as very low certainty. Risk of bias was judged serious because recurrent concerns remained regarding allocation concealment and outcome-assessor blinding in the randomized studies and because two quasi-experimental studies contributed to the pooled estimate. Inconsistency was judged very serious because heterogeneity was extreme (I^2^ = 94.70%; τ^2^ = 1.50748) and the 95% prediction interval (−1.036 to 4.003) crossed the null and covered a very broad range of plausible effects. Indirectness was judged serious because 9 of the 11 meta-analysis studies were conducted in East Asian settings and 8 of 11 involved university populations, limiting generalizability to broader school-age and international physical-education contexts. Imprecision was not rated down because the 95% confidence interval for the pooled estimate (0.738 to 2.230) remained entirely on the side of benefit, although the exact magnitude remained uncertain and no established minimally important standardized effect threshold was available for this heterogeneous outcome construct. Publication bias was not rated down: Egger’s test was not statistically significant (*p* = 0.176) and Begg’s test was borderline (*p* = 0.052), but the small number of studies limits the ability to exclude reporting bias. The resulting certainty rating therefore indicates very limited confidence that the pooled magnitude is close to the true effect across comparable settings. These findings are summarized in Table 4.

**Table 4:** GRADE summary of findings for the primary meta-analytic outcome.

Outcome / Evidence base	Effect estimate	GRADE domain judgments	Certainty and interpretation
Post-intervention motor-skill performance; 11 studies (9 randomized, 2 quasi-experimental)	Hedges’ g = 1.484 (95% CI 0.738 to 2.230); 95% prediction interval −1.036 to 4.003	Risk of bias: serious (−1); inconsistency: very serious (−2); indirectness: serious (−1); imprecision: not serious; publication bias: no downgrade	Very low certainty. The average effect was positive, but confidence in the pooled magnitude is very limited because important bias, heterogeneity, and generalizability concerns remain.

The GRADE rating is outcome-level and is not a restatement of the JBI study appraisal. The randomized-trial-only sensitivity analysis yielded g = 1.662 (95% CI [0.768, 2.556]), I^2^ = 95.45%, τ^2^ = 1.77549, and a 95% prediction interval of −1.098 to 4.422, indicating that exclusion of the two quasi-experimental studies did not resolve the extreme heterogeneity. The prediction interval was considered primarily under inconsistency rather than imprecision.

### 4.7 Sensitivity analysis and small-study effects

Leave-one-out analyses indicated that the average pooled estimate remained positive after removing any single study, ranging from g = 1.175 to g = 1.611. Rakha (2026) had the largest study-level effect (g = 4.918) and was the only study flagged by the prespecified influence criteria (studentized residual = 2.667; Cook’s distance = 0.622). Excluding Rakha (2026) reduced the pooled estimate from g = 1.484 to g = 1.175 (95% CI [0.679, 1.671]). Because this confidence interval remained entirely above zero, the positive direction of the average pooled effect was not dependent on this influential study. However, substantial heterogeneity persisted (I^2^ = 93.54%), and the corresponding 95% prediction interval remained wide and crossed the null (−0.394 to 2.744). Removal of Rakha (2026) therefore reduced the magnitude of the average effect but did not resolve the large between-study variation in true effects or the uncertainty about the effect that might be observed in a new comparable setting. Rakha (2026) was retained in the prespecified primary model and explicitly treated as influential in interpretation. A *post hoc* randomized-trial-only sensitivity analysis restricted the model to the nine studies classified as randomized trials and yielded g = 1.662 (95% CI [0.768, 2.556]), with I^2^ = 95.45%, τ^2^ = 1.77549, and a 95% prediction interval of −1.098 to 4.422. Thus, the two quasi-experimental studies did not account for either the positive direction of the average estimate or the extreme between-study heterogeneity.

The within-study correlation sensitivity analysis for Lin et al. (2025) produced stable pooled estimates across *r* = 0.30, *r* = 0.50, and *r* = 0.70. Because the retracted article was removed, no dependent outcomes from that study contributed to the revised main model. Publication-bias analyses were exploratory because the main model included only 11 studies. Egger’s regression test was not statistically significant (*p* = 0.176), and Begg’s rank-correlation test was borderline and exploratory (*p* = 0.052) (Begg and Mazumdar, 1994; Egger et al., 1997). These results should be interpreted cautiously because small-study-effect tests have limited power and unstable error rates with small numbers of studies. The funnel plot is presented in Supplementary Figure S1.

## 5 Discussion

### 5.1 Reconsidering feedback timing and precision in motor-skill performance and learning

After removing the retracted article, the meta-analysis supported a positive average association between AI-based real-time feedback and students’ post-intervention motor-skill performance. The pooled estimate was positive (g = 1.484, 95% CI [0.738, 2.230]), and the confidence interval remained above zero. The positive direction of the average effect also persisted after exclusion of the influential Rakha (2026) study (g = 1.175, 95% CI [0.679, 1.671]). These results support a positive average effect across the studies included in the quantitative synthesis. This statistical conclusion concerns post-intervention performance rather than durable motor learning, which requires delayed retention or transfer evidence. The observed average is consistent with feedback theories that emphasize timeliness, specificity, and actionable information, particularly when feedback is connected to the next practice attempt (Hattie and Timperley, 2007; Kluger and DeNisi, 1996; Shute, 2008; Sigrist et al., 2013). AI-based systems may support this process by capturing movement features during practice, making deviations visible, and returning corrective information within the same instructional or practice cycle.

A positive average effect, however, should not be interpreted as implying that AI-based feedback produces a similarly positive effect in every physical-education setting. Between-study heterogeneity was extreme, and the 95% prediction interval for the primary model ranged from −1.036 to 4.003. The prediction interval addresses a different question from the confidence interval: whereas the confidence interval describes uncertainty around the average pooled effect, the prediction interval describes the range of true effects that may plausibly occur across comparable settings. Its wide range and crossing of the null indicate that the magnitude, and potentially the direction, of the effect in a new setting may differ considerably from the positive average observed across the existing studies. Accordingly, the pooled estimate is best understood as the average of a highly heterogeneous distribution of true effects rather than as a stable or uniformly expected intervention effect. Given the small evidence base, very low certainty, and substantial contextual variability, claims about the effect expected in any particular setting should remain cautious until more rigorous and directly comparable studies are available.

Feedback timing alone is unlikely to explain the observed variation in motor-skill outcomes. Feedback also has to direct attention to task-relevant information without overloading learners or encouraging dependence on external cues (Wulf and Prinz, 2001; Wulf and Shea, 2002). Feedback must also be interpretable, aligned with the learning goal, and embedded in practice. AI can increase the frequency and specificity of feedback, but the pedagogical value of that feedback depends on how it changes students’ perception of error and subsequent movement correction.

### 5.2 The instructional mechanisms of AI-supported movement feedback

The included studies suggest that AI-supported feedback may influence motor-skill performance by externalizing movement information through visualization, automated scoring, mobile correction, and sensor-based cues (Hsia et al., 2024; Hsu et al., 2025; Huang et al., 2026; Lin et al., 2025; Ma et al., 2025a). These tools can make posture, timing, sequence, or movement deviations more observable and support comparison with target patterns (Colyer et al., 2018; Mathis et al., 2018; Sigrist et al., 2013).

However, AI feedback still requires pedagogical interpretation. Teachers remain important for prioritizing cues, explaining corrective actions, and integrating system outputs with progressive practice; this supports a teacher-AI collaboration model rather than a replacement model (Roll and Wylie, 2016; VanLehn, 2011).

### 5.3 Contextual variability in the effects of AI-based feedback

The effects of AI-based feedback likely vary across movement tasks. Closed or technique-oriented skills may be more amenable to automated feedback because performance criteria can be represented through posture, sequence, timing, or movement accuracy. This interpretation aligns with the challenge-point view that task difficulty, information load, and learner skill level shape how feedback affects learning (Guadagnoli and Lee, 2004). Yoga, dance, Baduanjin, badminton strokes, and similar skills provide relatively observable movement patterns that can be compared with target forms.

Open sport skills may be more difficult to support through automated feedback alone. Football, basketball, handball, and other interactive games involve tactical decisions, perceptual demands, opponent behavior, and context-dependent movement choices. AI feedback can still support technical components within these sports, but a movement that is technically correct in isolation may not always be effective in a dynamic game context. This may partly explain why effects differed across sport domains and outcome categories.

Educational level may also shape intervention effects, as the evidence base included university, primary, secondary, vocational, youth sport, and mixed settings (Li, 2025; Lin, 2024; Lin, 2026; Zhong, 2026). University students made up the largest subgroup in the evidence base, while primary and secondary settings were less represented. Older students may be better able to interpret abstract visual or numerical feedback, whereas younger learners may require more teacher mediation and simpler cues. The current evidence is insufficient to determine whether education level moderates the effect, so future studies should examine developmental fit more directly.

The substantial between-study heterogeneity is likely to reflect several overlapping sources rather than a single moderator. First, the included interventions differed in technological implementation, including computer vision or pose and motion recognition, intelligent feedback or teaching systems, wearable or sensor-based systems, and other AI-supported platforms. Second, feedback was delivered through different modalities and at different levels of temporal proximity to practice, including visual or video feedback, dashboards and scores, automated corrective feedback, and teacher-mediated or mixed feedback. Third, the studies covered heterogeneous motor-skill domains and educational contexts, ranging from relatively closed, technique-oriented tasks to more open and interactive sport skills, and from university populations to school-age and vocational learners. Fourth, outcome measures varied across movement quality, sport-specific skill performance, composite skill scores, and other performance indicators, which may produce different standardized effect magnitudes even when interventions share a broadly similar instructional function. Study design and methodological quality also varied, and the influence analysis showed that the exceptionally large estimate from Rakha (2026) increased the magnitude and dispersion of the pooled result, although substantial heterogeneity remained after its exclusion. The exploratory subgroup analyses therefore provide possible contextual explanations but do not establish definitive moderators. With only 11 study-level effects in the primary meta-analysis and small numbers within individual subgroups, formal meta-regression or extensive moderator testing would have been statistically unstable. Accordingly, the observed heterogeneity should be interpreted as evidence that the effect of AI-based real-time feedback is context-dependent and that future studies should directly test prespecified technological, pedagogical, learner, and task-level moderators.

### 5.4 Evidence quality, reporting transparency, and methodological constraints

The design-specific appraisal does not support describing the 11 meta-analysis studies as uniformly high quality. Across the randomized trials, participant and intervention-provider blinding was generally not feasible, allocation concealment was often insufficiently reported, and outcome-assessor blinding was frequently unclear. Quasi-experimental and narrative-only studies more often showed incomplete reporting of control structures, follow-up, outcome-measurement reliability, or statistical analysis. These limitations should be considered alongside the substantial heterogeneity when interpreting the pooled estimate and the broader evidence base. The unusually large study-level effect estimates should also be interpreted in light of design and measurement characteristics that may inflate or destabilize standardized effects. In several studies, participant and intervention-provider blinding was not feasible, assessor blinding was unclear, samples were modest, and some outcomes relied on evaluator-rated or composite performance measures; these features may contribute to upwardly biased or unstable estimates.

At the body-of-evidence level, GRADE rated certainty for the pooled post-intervention motor-skill performance outcome as very low. This rating is conceptually different from the statistical significance of the pooled mean and from the JBI appraisal of individual studies. The confidence interval supports a positive average effect across the included studies, whereas the very low GRADE certainty indicates limited confidence in the magnitude, generalizability, and stability of that effect across contexts. The rating was driven principally by serious risk-of-bias concerns, very serious inconsistency, and serious indirectness related to the concentration of evidence in East Asian and university settings. Thus, the positive pooled average should not be interpreted as high-certainty evidence of a consistently large benefit across physical-education contexts.

A recurring constraint was incomplete reporting of group-level quantitative data. This reporting gap also reflects a broader challenge in AI-in-education research: technical novelty is often described more fully than the instructional theory, measurement validity, or replicable outcome data needed for cumulative evidence (Chen et al., 2020b; Hwang et al., 2020). Several studies described improvements in motor skill outcomes but did not provide the means, standard deviations, sample sizes, or convertible statistics needed for effect-size calculation. This limits cumulative evidence because studies can appear relevant and promising while remaining unusable for quantitative synthesis. Transparent outcome reporting determines whether future reviews can estimate effects reliably.

Another concern is the diversity of outcome measures. Motor-skill outcomes were assessed through movement quality, sport-specific performance, composite skill scores, time-to-proficiency indicators, and other measures. This diversity reflects the breadth of physical education, but it also complicates interpretation. Stronger evidence would require clearer definitions of motor skill outcomes, more standardized measurement where possible, and explicit reporting of reliability or validity for assessment tools. The outcome audit identified a four-week retention assessment in Lu et al. (2025). Bouzid et al. (2026) described a retention phase, but the delay interval was insufficiently specified for classification as delayed retention evidence. No included study reported a clearly identifiable transfer outcome.

### 5.5 Designing teacher-AI collaborative feedback in physical education

The practical implication is not that AI should replace teachers. AI can support observation, measurement, visualization, and timely correction, while teachers retain responsibility for pedagogical judgment, motivation, task progression, safety, and adaptation to learners’ needs.

Implementation should therefore prioritize feedback design: outputs should be clear, actionable, age-appropriate, task-relevant, and adjustable in timing and specificity. AI is most useful when it makes teacher feedback more precise and scalable without weakening the instructional relationship.

### 5.6 Limitations and future research directions

This review has several limitations. The final systematic-review evidence base comprised 18 studies after exclusion of one retracted article identified during reference verification, one purely qualitative participatory-action-research study that did not meet the quantitative/mixed-methods eligibility criterion, and two CNKI reports (documented in Supplementary Table S2) that did not provide sufficient full-text evidence to verify the required empirical AI-feedback intervention and student motor-skill outcomes. First, the number of studies included in the meta-analysis was modest, and small-study-effect tests should be interpreted cautiously (Sterne et al., 2011). Second, heterogeneity was high, indicating that the pooled estimate should not be generalized without attention to context or to the likely range of effects in future settings (Higgins et al., 2003; Riley et al., 2011). Third, AI technologies, feedback formats, motor skill domains, and outcome measures varied substantially across studies. Because only 11 study-level effects entered the primary meta-analysis and the corresponding subgroups were small, formal meta-regression was not undertaken; subgroup findings were therefore interpreted as exploratory rather than as evidence of definitive moderators. Fourth, several studies lacked complete quantitative data, which limited their contribution to the meta-analysis. Geographical concentration was also substantial: 16 of the 18 included studies (88.9%) were conducted in mainland China or Taiwan. Consequently, the findings may reflect specific educational, technological, and institutional contexts and should not be assumed to generalize to physical-education systems in other regions.

Future research should use stronger experimental designs, including adequately powered randomized or well-controlled quasi-experimental studies. Studies should report group sample sizes, means, standard deviations, attrition, baseline equivalence, and reliability of motor skill measures. Researchers should also compare different feedback designs, such as immediate corrective feedback, visual and video feedback, dashboard feedback, and teacher-mediated AI feedback, rather than treating AI feedback as a single intervention category.

Longer follow-up is also needed. Most studies evaluated immediate or post-intervention motor-skill performance, but motor skill learning should ideally be assessed through retention, transfer, and application in realistic physical education contexts. Future work should examine whether AI-supported feedback produces durable learning, how students use feedback over time, and how teachers can integrate AI outputs into inclusive and developmentally appropriate instruction.

## 6 Conclusion

Current evidence supports a positive average effect of AI-based real-time feedback on post-intervention motor-skill performance in physical education. The primary pooled confidence interval remained above zero, and the positive direction of the average effect persisted after removal of the influential Rakha (2026) study. However, between-study heterogeneity was extreme and the 95% prediction interval crossed the null, indicating that the magnitude and direction of the effect expected in an individual future setting remain considerably less certain. The pooled estimate should therefore be understood as an average across highly heterogeneous true effects rather than as a stable or consistently reproducible intervention effect across physical-education contexts. GRADE certainty for the pooled outcome was very low because of serious risk-of-bias concerns, very serious inconsistency, and serious indirectness; this limits confidence in the magnitude and generalizability of the effect without negating the positive average observed in the current meta-analysis. Because the primary meta-analysis synthesized post-intervention performance and did not include delayed retention or transfer outcomes, the present review also cannot determine whether any observed performance advantage represents durable motor learning. More rigorous, adequately powered studies with comparable outcome definitions and delayed retention and transfer assessments are needed before firm conclusions regarding effects across specific physical-education settings can be drawn.

## Data Availability

The original contributions presented in the study are included in the article/Supplementary material, further inquiries can be directed to the corresponding authors.
