# Supplementary material for: Effects of AI-based real-time feedback on students’ motor-skill performance in physical education: a systematic review and meta-analysis
Source: Front Psychol. 2026 Oct 28;17:1946151. doi: 10.3389/fpsyg.2026.1946151 (PMC13751374; doi:10.3389/fpsyg.2026.1946151)
Supplement: Supplementary file 1 [file Supplementary_file_1.DOCX]

Supplementary Information

# README

| Item | Description |
| --- | --- |
| Scope | Final supplementary material after removal of one retracted article, exclusion of one ineligible qualitative study, and independent eligibility reassessment excluding Bi (2025) and Han (2025). Zhong (2026) remains included for narrative synthesis. |
| Final counts | Systematic review n=18; meta-analysis n=11 study-level effects. |
| **Certainty of evidence** | Supplementary Table S5 provides the GRADE evidence profile for the primary post-intervention motor-skill performance outcome. |

# S1 Search Strategy

| **Database / round** | **Full search strategy** | **Date / limits / records** | **Documentation** |
| --- | --- | --- | --- |
| Web of Science | (("artificial intelligence" OR AI OR "machine learning" OR "deep learning" OR "computer vision" OR "pose estimation" OR "motion capture" OR "wearable sensor*" OR "smart system*" OR "intelligent system*" OR "automated system*" OR "intelligent feedback") AND ("real-time feedback" OR "real time feedback" OR "immediate feedback" OR "instant feedback" OR "augmented feedback" OR "automated feedback" OR "visual feedback" OR "video feedback" OR "corrective feedback" OR "personalized feedback" OR feedback) AND ("physical education" OR "school physical education" OR "PE class*" OR "physical education teaching" OR "physical education lesson*" OR "motor skill*" OR "movement skill*" OR "fundamental movement skill*" OR "fundamental motor skill*" OR "motor learning" OR "skill acquisition")) | Journal articles; 2014-01-01 to 2026-06-25 | Search strategy and eligibility criteria file; no local path retained in final supplement. |
| ERIC | ("artificial intelligence" OR AI OR "machine learning" OR "deep learning" OR "computer vision" OR "pose estimation" OR "automated feedback" OR "intelligent feedback" OR "wearable sensor") AND ("feedback" OR "real-time feedback" OR "real time feedback" OR "immediate feedback" OR "augmented feedback" OR "visual feedback" OR "video feedback" OR "corrective feedback") AND ("physical education" OR "school physical education" OR "motor skills" OR "motor learning" OR "movement skills" OR "fundamental motor skills" OR "skill acquisition") | Journal articles; 2014-01-01 to 2026-06-25 | Search strategy and eligibility criteria file; no local path retained in final supplement. |
| **PubMed — Search 1** | (("Artificial Intelligence"[Mesh] OR "Machine Learning"[Mesh] OR "artificial intelligence"[Title/Abstract] OR AI[Title/Abstract] OR "machine learning"[Title/Abstract] OR "deep learning"[Title/Abstract] OR "computer vision"[Title/Abstract] OR "pose estimation"[Title/Abstract] OR "motion capture"[Title/Abstract] OR "wearable sensor*"[Title/Abstract] OR "automated feedback"[Title/Abstract] OR "intelligent system*"[Title/Abstract]) AND ("Feedback"[Mesh] OR feedback[Title/Abstract] OR "real-time feedback"[Title/Abstract] OR "real time feedback"[Title/Abstract] OR "immediate feedback"[Title/Abstract] OR "instant feedback"[Title/Abstract] OR "augmented feedback"[Title/Abstract] OR "visual feedback"[Title/Abstract] OR "video feedback"[Title/Abstract] OR "corrective feedback"[Title/Abstract] OR "personalized feedback"[Title/Abstract]) AND ("Physical Education and Training"[Mesh] OR "Motor Skills"[Mesh] OR "Psychomotor Performance"[Mesh] OR "physical education"[Title/Abstract] OR "school physical education"[Title/Abstract] OR "PE class*"[Title/Abstract] OR "motor skill*"[Title/Abstract] OR "motor learning"[Title/Abstract] OR "movement skill*"[Title/Abstract] OR "fundamental motor skill*"[Title/Abstract] OR "skill acquisition"[Title/Abstract]) AND ("Students"[Mesh] OR "Child"[Mesh] OR "Adolescent"[Mesh] OR student*[Title/Abstract] OR pupil*[Title/Abstract] OR child*[Title/Abstract] OR adolescent*[Title/Abstract] OR youth[Title/Abstract] OR learner*[Title/Abstract])) AND 2014/01/01:2026/06/25[Date - Publication] | Actual final search/export date: 25 June 2026. Initial eligibility period began: 1 January 2014. Author-confirmed original publication-date limit: 2014/01/01:2026/06/25[Date - Publication]. Records exported: 96. Journal Article / English / Humans filter activation not independently verified. | Recovered strategy record (26 June 2026), with publication-date cutoff corrected based on author confirmation; exports dated 25 June 2026. |
| **PubMed — Search 2** | (("artificial intelligence"[Title/Abstract] OR AI[Title/Abstract] OR "machine learning"[Title/Abstract] OR "deep learning"[Title/Abstract] OR "computer vision"[Title/Abstract] OR "pose estimation"[Title/Abstract] OR "motion capture"[Title/Abstract] OR "wearable sensor*"[Title/Abstract] OR "automated feedback"[Title/Abstract] OR "intelligent feedback"[Title/Abstract]) AND ("feedback"[Title/Abstract] OR "real-time feedback"[Title/Abstract] OR "real time feedback"[Title/Abstract] OR "immediate feedback"[Title/Abstract] OR "augmented feedback"[Title/Abstract] OR "visual feedback"[Title/Abstract] OR "video feedback"[Title/Abstract] OR "corrective feedback"[Title/Abstract]) AND ("physical education"[Title/Abstract] OR "school physical education"[Title/Abstract] OR "PE class*"[Title/Abstract] OR "physical education teaching"[Title/Abstract] OR "physical education lesson*"[Title/Abstract]) AND (student*[Title/Abstract] OR pupil*[Title/Abstract] OR child*[Title/Abstract] OR adolescent*[Title/Abstract] OR youth[Title/Abstract] OR learner*[Title/Abstract])) AND 2014/01/01:2026/06/25[Date - Publication] | Actual final search/export date: 25 June 2026. Initial eligibility period began: 1 January 2014. Author-confirmed original publication-date limit: 2014/01/01:2026/06/25[Date - Publication]. Records exported: 17. Journal Article / English / Humans filter activation not independently verified. | Recovered strategy record (26 June 2026), with publication-date cutoff corrected based on author confirmation; exports dated 25 June 2026. |
| **PubMed — Search 3** | (("computer vision"[Title/Abstract] OR "pose estimation"[Title/Abstract] OR "motion capture"[Title/Abstract] OR "wearable sensor*"[Title/Abstract] OR "machine learning"[Title/Abstract] OR "deep learning"[Title/Abstract] OR "artificial intelligence"[Title/Abstract] OR AI[Title/Abstract]) AND ("feedback"[Title/Abstract] OR "real-time feedback"[Title/Abstract] OR "real time feedback"[Title/Abstract] OR "immediate feedback"[Title/Abstract] OR "automated feedback"[Title/Abstract] OR "augmented feedback"[Title/Abstract]) AND ("motor skill*"[Title/Abstract] OR "motor learning"[Title/Abstract] OR "movement skill*"[Title/Abstract] OR "fundamental motor skill*"[Title/Abstract] OR "skill acquisition"[Title/Abstract]) AND (student*[Title/Abstract] OR child*[Title/Abstract] OR adolescent*[Title/Abstract] OR youth[Title/Abstract] OR "physical education"[Title/Abstract])) NOT (surgery[Title/Abstract] OR surgical[Title/Abstract] OR laparoscopic[Title/Abstract] OR "medical education"[Title/Abstract] OR rehabilitation[Title/Abstract] OR patient*[Title/Abstract]) AND 2014/01/01:2026/06/25[Date - Publication] | Actual final search/export date: 25 June 2026. Initial eligibility period began: 1 January 2014. Author-confirmed original publication-date limit: 2014/01/01:2026/06/25[Date - Publication]. Records exported: 19. Journal Article / English / Humans filter activation not independently verified. | Recovered strategy record (26 June 2026), with publication-date cutoff corrected based on author confirmation; exports dated 25 June 2026. |
| **CNKI — Search 1** | Documented Subject-field strategy: 主题 = （人工智能 + 机器学习 + 深度学习 + 计算机视觉 + 姿态识别 + 动作识别 + 智能反馈） AND 主题 = （实时反馈 + 即时反馈 + 自动反馈 + 纠错反馈 + 视觉反馈 + 视频反馈 + 个性化反馈） AND 主题 = （体育教学 + 体育教育 + 学校体育 + 体育课 + 运动技能 + 动作技能 + 技能学习）  Explicit Boolean interpretation (OR within concept groups; AND between groups; field: Subject/主题; interface syntax not independently verified): 主题 = (人工智能 OR 机器学习 OR 深度学习 OR 计算机视觉 OR 姿态识别 OR 动作识别 OR 智能反馈) AND 主题 = (实时反馈 OR 即时反馈 OR 自动反馈 OR 纠错反馈 OR 视觉反馈 OR 视频反馈 OR 个性化反馈) AND 主题 = (体育教学 OR 体育教育 OR 学校体育 OR 体育课 OR 运动技能 OR 动作技能 OR 技能学习) | Recorded search/export date: 25 June 2026. Recorded publication window: 1 January 2014–25 June 2026. Records exported: 28. Only Chinese journal articles were eligible for screening; exports 1 and 3 included non-journal records. | Archived strategy file (26 June 2026); CNKI screening workbook (29 June 2026). |
| **CNKI — Search 2** | Documented Subject-field strategy: 主题 = （智慧体育 + 智能体育 + 数字体育 + 体育智能化 + 人工智能体育 + AI体育） AND 主题 = （反馈 + 评价 + 纠错 + 指导 + 教学辅助 + 动作评价） AND 主题 = （学生 + 中小学生 + 大学生 + 体育课 + 体育教学 + 运动技能）  Explicit Boolean interpretation (OR within concept groups; AND between groups; field: Subject/主题; interface syntax not independently verified): 主题 = (智慧体育 OR 智能体育 OR 数字体育 OR 体育智能化 OR 人工智能体育 OR AI体育) AND 主题 = (反馈 OR 评价 OR 纠错 OR 指导 OR 教学辅助 OR 动作评价) AND 主题 = (学生 OR 中小学生 OR 大学生 OR 体育课 OR 体育教学 OR 运动技能) | Recorded search/export date: 25 June 2026. Recorded publication window: 1 January 2014–25 June 2026. Records exported: 362. Only Chinese journal articles were eligible for screening; exports 1 and 3 included non-journal records. | Archived strategy file (26 June 2026); CNKI screening workbook (29 June 2026). |
| **CNKI — Search 3** | Documented Subject-field strategy: 主题 = （姿态识别 + 动作识别 + 动作捕捉 + 人体姿态估计 + 运动识别 + 骨骼点识别） AND 主题 = （反馈 + 纠错 + 评价 + 指导 + 动作分析 + 动作质量） AND 主题 = （体育教学 + 体育课 + 运动技能 + 动作学习 + 武术 + 太极 + 篮球 + 体操）  Explicit Boolean interpretation (OR within concept groups; AND between groups; field: Subject/主题; interface syntax not independently verified): 主题 = (姿态识别 OR 动作识别 OR 动作捕捉 OR 人体姿态估计 OR 运动识别 OR 骨骼点识别) AND 主题 = (反馈 OR 纠错 OR 评价 OR 指导 OR 动作分析 OR 动作质量) AND 主题 = (体育教学 OR 体育课 OR 运动技能 OR 动作学习 OR 武术 OR 太极 OR 篮球 OR 体操) | Recorded search/export date: 25 June 2026. Recorded publication window: 1 January 2014–25 June 2026. Records exported: 111. Only Chinese journal articles were eligible for screening; exports 1 and 3 included non-journal records. | Archived strategy file (26 June 2026); CNKI screening workbook (29 June 2026). |

Search date and date coverage. Searches and bibliographic exports for the three PubMed rounds were completed on 25 June 2026. The review team confirms the original PubMed publication-date upper cutoff was also 25 June 2026; each reported search expression uses 2014/01/01:2026/06/25[Date - Publication]. The initial lower publication-date limit (2014) is addressed separately in the review methodology and Reviewer 4 Comment 2B.

Search-strategy documentation. The complete expressions were recovered from project search-strategy records compiled after the searches and were cross-checked against dated bibliographic exports and the CNKI screening workbook. The PubMed date limits in this supplement reflect the original cutoff confirmed by the review team. The preserved materials did not contain database-generated PubMed Search Details or CNKI search-history screenshots; therefore, additional interface settings not established by those records are not represented as applied.

PubMed filters and counts. The project documentation mentions Journal Article, English, and Humans as possible PubMed filters, but their activation during each of the three searches could not be independently established. These are not claimed as applied search filters. The 96, 17, and 19 figures are round-specific exported-record counts before within-PubMed deduplication; they should not be added as distinct-study counts.

CNKI search notation and record types. The historical notes specified the Subject (主题) field, a “+” notation joining alternatives within a concept group, and AND between concept groups. The expanded OR/AND version makes the intended conceptual logic explicit but is not claimed to be a verbatim export from CNKI’s advanced-search interface. Search exports contained 28, 362, and 111 records, respectively, including non-journal material. Non-journal records were removed after export, before journal-only title/abstract screening; no universal journal-only interface filter is claimed.

# S2 Full Text Exclusion Reasons

| Screening stage | Source | Reason category | Count | Notes |
| --- | --- | --- | --- | --- |
| Records removed before screening | CNKI | non-journal publications removed after export, before journal-only title/abstract screening | 100 | CNKI theses, dissertations, conference papers, books, newspapers, patents and standards removed before journal-only screening. |
| Title/abstract exclusion | WOS/ERIC/PubMed | did not meet title/abstract eligibility criteria | 388 | After deduplication, title/abstract screening excluded 388 records. |
| Title/abstract exclusion | CNKI journal-only | did not meet title/abstract eligibility criteria | 337 | CNKI journal-only title/abstract screening excluded 337 records. |
| Not retrieved | WOS/ERIC/PubMed | full text unavailable | 6 | Reports sought but not retrieved. |
| Full-text exclusion | WOS/ERIC/PubMed | book / not journal empirical article | 1 | Book/editorial volume excluded as not an empirical journal article. |
| Full-text exclusion | WOS/ERIC/PubMed | no AI-based or intelligent feedback component | 4 | Counts are not mutually exclusive where multiple exclusion codes were assigned. |
| Full-text exclusion | WOS/ERIC/PubMed | no real-time or automated feedback | 5 | Counts are not mutually exclusive where multiple exclusion codes were assigned. |
| Full-text exclusion | WOS/ERIC/PubMed | not student PE or educational motor-skill context | 8 | Counts are not mutually exclusive where multiple exclusion codes were assigned. |
| Full-text exclusion | WOS/ERIC/PubMed | not target population/context | 1 | Counts are not mutually exclusive where multiple exclusion codes were assigned. |
| Full-text exclusion | WOS/ERIC/PubMed | theoretical / non-empirical / pathway or model discussion | 4 | Counts are not mutually exclusive where multiple exclusion codes were assigned. |
| Full-text exclusion | WOS/ERIC/PubMed | no motor-skill outcome | 20 | Counts are not mutually exclusive where multiple exclusion codes were assigned. |
| Full-text exclusion | WOS/ERIC/PubMed | insufficient full-text information | 1 | Counts are not mutually exclusive where multiple exclusion codes were assigned. |
| Full-text exclusion | CNKI | no AI-based or intelligent feedback component | 2 | Counts are not mutually exclusive where multiple exclusion codes were assigned. |
| Full-text exclusion | CNKI | no real-time or automated feedback | 2 | Counts are not mutually exclusive where multiple exclusion codes were assigned. |
| Full-text exclusion | CNKI | not student PE or educational motor-skill context | 4 | Counts are not mutually exclusive where multiple exclusion codes were assigned. |
| Full-text exclusion | CNKI | theoretical / non-empirical / pathway or model discussion | 38 | Counts are not mutually exclusive where multiple exclusion codes were assigned. |
| Full-text/revision eligibility exclusion | CNKI | insufficient evidence to verify an implemented AI-based feedback intervention and student motor-skill outcomes | 2 | Bi, W. (2025), “Research and practice on an AI-based smart classroom teaching model for secondary vocational physical education,” Sporting Goods and Technology, 2025(19), 167–170; and Han, X. (2025), “Construction and practical pathways of a generative AI-driven intelligent teaching model for general college physical education,” Sporting Goods and Technology, 2025(12), 183–186. Both excluded after independent two-reviewer full-text eligibility reassessment. Exclusion was not based on absence of a DOI or effect-size SD. |
| Full-text exclusion | All sources | total reports excluded after eligibility assessment | 78 | WOS/ERIC/PubMed n=29; CNKI n=47 (including Bi 2025 and Han 2025); book/not journal n=1; retracted n=1. |
| Full-text exclusion | CNKI | no motor-skill outcome | 6 | Counts are not mutually exclusive where multiple exclusion codes were assigned. |
| Full-text/revision eligibility exclusion | WOS/ERIC/PubMed | purely qualitative study without quantitative or mixed-methods student-level motor-skill outcome data | 1 | Kim_2026; excluded after independent eligibility re-verification during revision. |
| Full-text/reference verification | WOS/ERIC/PubMed | Retracted article | 1 | Liu_2024; DOI 10.1007/s11082-023-06243-0; Retraction note DOI 10.1007/s11082-024-07600-3. |

# S3 Effect Size Extraction Details

| Study (author–year) | Outcome name | Outcome category | n_E | Mean_E | SD_E | n_C | Mean_C | SD_C | Hedges’ g | Variance | Data extraction method | Included in main model | Reason if not included | Page/table source | Row type |
| --- | --- | --- | --- | --- | --- | --- | --- | --- | --- | --- | --- | --- | --- | --- | --- |
| Bouzid et al. (2026) | Soccer tackling trunk-leg angle | Movement quality / technique execution | 12 | 145.9 | 14.53 | 11 | 120.54 | 11.94 | 1.829671 | 0.253949 | post-test mean SD | No | Raw result-level effect; main model used one study-level effect per study. | PDF page 5, Table 1 | Raw result-level effect |
| Bouzid et al. (2026) | Overall soccer tackling technical performance score | Sport-specific skill performance | 12 | NR | NR | 11 | NR | NR | NR | NR | reported effect size | No | Raw result-level effect; main model used one study-level effect per study. | PDF page 5, Figure 4A/text | Raw result-level effect |
| Bouzid et al. (2026) | Soccer tackling criterion C3: correct positioning of leg and knee behind the ball | Movement quality / technique execution | 12 | NR | NR | 11 | NR | NR | NR | NR | reported effect size | No | Raw result-level effect; main model used one study-level effect per study. | PDF page 5, Figure 4B/text | Raw result-level effect |
| Bouzid et al. (2026) | Soccer tackling criterion C4: ball contact | Movement quality / technique execution | 12 | NR | NR | 11 | NR | NR | NR | NR | reported effect size | No | Raw result-level effect; main model used one study-level effect per study. | PDF page 5, Figure 4C/text | Raw result-level effect |
| Huang et al. (2026) | Yogism movement skills performance | Movement quality / technique execution | 40 | 2.46 | 0.33 | 40 | 2.19 | 0.25 | 0.913406 | 0.055348 | post-test mean SD | No | Raw result-level effect; main model used one study-level effect per study. | PDF page 14, Table 2 | Raw result-level effect |
| Lin (2026) | 技能考核成绩 / Skill test score | Sport-specific skill performance | 108 | 89.5 | 4.2 | 108 | 82.1 | 5.7 | 1.472893 | 0.023587 | post-test mean SD | No | Raw result-level effect; main model used one study-level effect per study. | PDF page 4, 表1 实验组与对照组教学效果后测对比分析 | Raw result-level effect |
| Lin (2026) | 战术应用提升幅度 / Tactical application improvement | Sport-specific skill performance | 108 | 显著更高 | NR | 108 | NR | NR | NR | NR | manual check required | No | Raw result-level effect; main model used one study-level effect per study. | PDF page 4, 表1 | Raw result-level effect |
| Lin (2026) | 动作标准性提升幅度 / Action standardization improvement | Movement quality / technique execution | 108 | 显著更高 | NR | 108 | NR | NR | NR | NR | manual check required | No | Raw result-level effect; main model used one study-level effect per study. | PDF page 4, 表1 | Raw result-level effect |
| Rakha (2026) | Handball passing | Sport-specific skill performance | 28 | 2.92 | 0.3 | 28 | 2.36 | 0.18 | 2.23208 | 0.11756 | post-test mean SD | No | Raw result-level effect; main model used one study-level effect per study. | PDF page 9, Table 3 | Raw result-level effect |
| Rakha (2026) | Handball catching | Sport-specific skill performance | 28 | 2.89 | 0.36 | 28 | 2.29 | 0.19 | 2.055429 | 0.110547 | post-test mean SD | No | Raw result-level effect; main model used one study-level effect per study. | PDF page 9, Table 3 | Raw result-level effect |
| Rakha (2026) | Handball dribbling | Sport-specific skill performance | 28 | 2.91 | 0.29 | 28 | 2.35 | 0.2 | 2.216739 | 0.116928 | post-test mean SD | No | Raw result-level effect; main model used one study-level effect per study. | PDF page 9, Table 3 | Raw result-level effect |
| Rakha (2026) | Handball shooting | Sport-specific skill performance | 28 | 2.85 | 0.33 | 28 | 2.39 | 0.2 | 1.662352 | 0.097016 | post-test mean SD | No | Raw result-level effect; main model used one study-level effect per study. | PDF page 9, Table 3 | Raw result-level effect |
| Rakha (2026) | Handball faking | Sport-specific skill performance | 28 | 2.76 | 0.29 | 28 | 2.42 | 0.2 | 1.345877 | 0.088201 | post-test mean SD | No | Raw result-level effect; main model used one study-level effect per study. | PDF page 9, Table 3 | Raw result-level effect |
| Rakha (2026) | Handball defense | Sport-specific skill performance | 28 | 2.87 | 0.26 | 28 | 2.37 | 0.18 | 2.204867 | 0.116442 | post-test mean SD | No | Raw result-level effect; main model used one study-level effect per study. | PDF page 9, Table 3 | Raw result-level effect |
| Rakha (2026) | Handball goalkeeping | Sport-specific skill performance | 28 | 2.77 | 0.39 | 28 | 2.35 | 0.13 | 1.424683 | 0.090222 | post-test mean SD | No | Raw result-level effect; main model used one study-level effect per study. | PDF page 9, Table 3 | Raw result-level effect |
| Rakha (2026) | Handball composite skill score | Physical skill proficiency | 28 | 2.85 | 0.12 | 28 | 2.36 | 0.07 | 4.918468 | 0.295422 | post-test mean SD | No | Raw result-level effect; main model used one study-level effect per study. | PDF page 9, Table 3 | Raw result-level effect |
| Sun and Lin (2026) | Obstacle dribble three-step layup (basketball) | Sport-specific skill performance | 58 | NR | NR | 56 | NR | NR | NR | NR | reported effect size | No | Raw result-level effect; main model used one study-level effect per study. | PDF pages 14-15, Table 4 | Raw result-level effect |
| Sun and Lin (2026) | Dribble shot (soccer) | Sport-specific skill performance | 58 | NR | NR | 56 | NR | NR | NR | NR | reported effect size | No | Raw result-level effect; main model used one study-level effect per study. | PDF pages 14-15, Table 4 | Raw result-level effect |
| Sun and Lin (2026) | Forehand and backhand reception (tennis) | Sport-specific skill performance | 58 | NR | NR | 56 | NR | NR | NR | NR | reported effect size | No | Raw result-level effect; main model used one study-level effect per study. | PDF pages 14-15, Table 4 | Raw result-level effect |
| Hsia et al. (2025) | Yoga performance | Movement quality / technique execution | 45 | 3.02 | 0.34 | 43 | 2.5 | 0.36 | 1.473097 | 0.058094 | post-test mean SD | No | Raw result-level effect; main model used one study-level effect per study. | PDF page 14, Table 1 | Raw result-level effect |
| Hsu et al. (2025) | Badminton clear distance score | Sport-specific skill performance | 67 | 13.68 | 3.11 | 71 | 10.47 | 4.35 | 0.840264 | 0.031606 | post-test mean SD | No | Raw result-level effect; main model used one study-level effect per study. | PDF page 12, clear-distance results; Table 9 referenced | Raw result-level effect |
| Hsu et al. (2025) | Expert manual badminton clear skill score | Movement quality / technique execution | 67 | NR | NR | 71 | NR | NR | NR | NR | manual check required | No | Raw result-level effect; main model used one study-level effect per study. | PDF page 12, Table 10 referenced | Raw result-level effect |
| Lin et al. (2025) | Badminton serve performance | Sport-specific skill performance | 45 | 66.52 | 12.34 | 44 | 59.9 | 10.65 | 0.568909 | 0.04681 | post-test mean SD | No | Raw result-level effect; main model used one study-level effect per study. | PDF page 14, Tables 2-3 | Raw result-level effect |
| Lin et al. (2025) | Badminton clear performance | Sport-specific skill performance | 49 | 61.36 | 10.82 | 47 | 55.13 | 11.43 | 0.555635 | 0.043327 | post-test mean SD | No | Raw result-level effect; main model used one study-level effect per study. | PDF page 15, Tables 2 and 4 | Raw result-level effect |
| Lu et al. (2025) | Time to proficiency (hours) | Physical skill proficiency | 100 | 31.3 | 4.1 | 100 | 48.2 | 6.3 | 3.167577 | 0.045337 | post-test mean SD | No | Raw result-level effect; main model used one study-level effect per study. | PDF page 20, Table 16 | Raw result-level effect |
| Lu et al. (2025) | Final cheerleading skill score | Sport-specific skill performance | 100 | 8.6 | 0.6 | 100 | 6.5 | 0.8 | 2.958585 | 0.042104 | post-test mean SD | No | Raw result-level effect; main model used one study-level effect per study. | PDF page 20, Table 16 | Raw result-level effect |
| Lu et al. (2025) | Retention rate at 4 weeks | Physical skill proficiency | 100 | 89.3% | NR | 100 | 71.2% | NR | NR | NR | manual check required | No | Raw result-level effect; main model used one study-level effect per study. | PDF page 20, Table 16 | Raw result-level effect |
| Ma et al. (2025a) | Baduanjin movement specifications | Movement quality / technique execution | 28 | 3.47 | 0.35 | 32 | 3.27 | 0.35 | 0.564007 | 0.069707 | post-test mean SD | No | Raw result-level effect; main model used one study-level effect per study. | PDF page 11, Table 3 | Raw result-level effect |
| Ma et al. (2025a) | Baduanjin demonstration level | Movement quality / technique execution | 28 | 3.45 | 0.39 | 32 | 3.21 | 0.37 | 0.624294 | 0.070324 | post-test mean SD | No | Raw result-level effect; main model used one study-level effect per study. | PDF page 11, Table 3 | Raw result-level effect |
| Ma et al. (2025a) | Baduanjin total score | Physical skill proficiency | 28 | 6.92 | 0.71 | 32 | 6.48 | 0.7 | 0.616294 | 0.070239 | post-test mean SD | No | Raw result-level effect; main model used one study-level effect per study. | PDF page 11, Table 3 | Raw result-level effect |
| Xu et al. (2025) | Dance skills performance | Movement quality / technique execution | 19 | 14.316 | 3.4 | 21 | 11.333 | 2.556 | 0.979253 | 0.112868 | post-test mean SD | No | Raw result-level effect; main model used one study-level effect per study. | PDF page 8, Table 3 | Raw result-level effect |
| Hsia et al. (2024) | Yoga skills performance | Movement quality / technique execution | 96 | 3.12 | 0.31 | 91 | 3.04 | 0.29 | 0.265198 | 0.021596 | post-test mean SD | No | Raw result-level effect; main model used one study-level effect per study. | Table 3, ANCOVA results of yoga skills performance | Raw result-level effect |
| Bouzid et al. (2026) | Soccer tackling trunk-leg angle | Movement quality / technique execution | NR | NR | NR | NR | NR | NR | 1.829671 | 0.253949 | Study-level effect used in main model | Yes |  | Computed from extracted result-level data or retained direct study-level effect. | Final study-level effect |
| Huang et al. (2026) | Yogism movement skills performance | Movement quality / technique execution | NR | NR | NR | NR | NR | NR | 0.913406 | 0.055348 | Study-level effect used in main model | Yes |  | Computed from extracted result-level data or retained direct study-level effect. | Final study-level effect |
| Lin (2026) | 技能考核成绩 / Skill test score | Sport-specific skill performance | NR | NR | NR | NR | NR | NR | 1.472893 | 0.023587 | Study-level effect used in main model | Yes |  | Computed from extracted result-level data or retained direct study-level effect. | Final study-level effect |
| Rakha (2026) | Handball composite skill score | Sport-specific skill performance | NR | NR | NR | NR | NR | NR | 4.918468 | 0.295422 | Study-level effect used in main model | Yes |  | Computed from extracted result-level data or retained direct study-level effect. | Final study-level effect |
| Hsia et al. (2025) | Yoga performance | Movement quality / technique execution | NR | NR | NR | NR | NR | NR | 1.473097 | 0.058094 | Study-level effect used in main model | Yes |  | Computed from extracted result-level data or retained direct study-level effect. | Final study-level effect |
| Hsu et al. (2025) | Badminton clear distance score | Sport-specific skill performance | NR | NR | NR | NR | NR | NR | 0.840264 | 0.031606 | Study-level effect used in main model | Yes |  | Computed from extracted result-level data or retained direct study-level effect. | Final study-level effect |
| Lin et al. (2025) | Composite of multiple dependent motor-skill outcomes | Sport-specific skill performance | NR | NR | NR | NR | NR | NR | 0.562272 | 0.033793 | Study-level effect used in main model | Yes |  | Computed from extracted result-level data or retained direct study-level effect. | Final study-level effect |
| Lu et al. (2025) | Final cheerleading skill score | Sport-specific skill performance | NR | NR | NR | NR | NR | NR | 2.958585 | 0.042104 | Study-level effect used in main model | Yes |  | Computed from extracted result-level data or retained direct study-level effect. | Final study-level effect |
| Ma et al. (2025a) | Baduanjin total score | Other | NR | NR | NR | NR | NR | NR | 0.616294 | 0.070239 | Study-level effect used in main model | Yes |  | Computed from extracted result-level data or retained direct study-level effect. | Final study-level effect |
| Xu et al. (2025) | Dance skills performance | Movement quality / technique execution | NR | NR | NR | NR | NR | NR | 0.979253 | 0.112868 | Study-level effect used in main model | Yes |  | Computed from extracted result-level data or retained direct study-level effect. | Final study-level effect |
| Hsia et al. (2024) | Yoga skills performance | Movement quality / technique execution | NR | NR | NR | NR | NR | NR | 0.265198 | 0.021596 | Study-level effect used in main model | Yes |  | Computed from extracted result-level data or retained direct study-level effect. | Final study-level effect |

Outcome-timing note. Lu et al. (2025) reported a four-week retention assessment. Bouzid et al. (2026) described a retention phase, but the delay interval was insufficiently specified for classification as delayed retention evidence. Neither retention-related result entered the primary meta-analysis, and no included study reported a clearly identifiable transfer outcome. Study labels have been converted from internal identifiers to author–year citations corresponding to the References section. The raw outcome-level effects and final study-level effects are displayed in separate blocks; within each block, studies are ordered from newest to oldest and alphabetically within year. Outcome names and row types retain traceability after removal of the Outcome_ID column.

# S4 Risk-of-Bias and Methodological Appraisal Details The detailed appraisal tables identify the assessed studies by author–year references matching the main manuscript and use reverse chronological order within each design group.

**Table S4a. Randomized controlled trials (revised JBI RCT tool).**

| **Study (author–year)** | **Synthesis role** | **Final design** | **Q1** | **Q2** | **Q3** | **Q4** | **Q5** | **Q6** | **Q7** | **Q8** | **Q9** | **Q10** | **Q11** | **Q12** | **Q13** | **Key concerns** |
| --- | --- | --- | --- | --- | --- | --- | --- | --- | --- | --- | --- | --- | --- | --- | --- | --- |
| Bouzid et al. (2026) | Primary meta-analysis | Randomized controlled trial | Yes | Unclear | Yes | No | No | Yes | Yes | Yes | Yes | Yes | Yes | Yes | Yes | Q2 Unclear, Q4 No, Q5 No |
| Lin (2026) | Primary meta-analysis | Randomized controlled trial as reported | Unclear | Unclear | Yes | No | No | Yes | Unclear | Yes | Unclear | Unclear | Unclear | Yes | Yes | Q1 Unclear, Q2 Unclear, Q4 No, Q5 No, Q7 Unclear, Q9 Unclear, Q10 Unclear, Q11 Unclear |
| Rakha (2026) | Primary meta-analysis | Randomized controlled trial | Yes | Unclear | Yes | No | No | Yes | Yes | Yes | Yes | Yes | Yes | Yes | Yes | Q2 Unclear, Q4 No, Q5 No |
| Hsia et al. (2025) | Primary meta-analysis | Class-level/cluster randomized controlled trial | Yes | Unclear | Yes | No | No | Yes | Unclear | Yes | Yes | Yes | Yes | Yes | Yes | Q2 Unclear, Q4 No, Q5 No, Q7 Unclear |
| Hsu et al. (2025) | Primary meta-analysis | Class-level/cluster randomized controlled trial | Yes | Unclear | Yes | No | No | Yes | Unclear | Yes | Yes | Yes | Yes | Yes | Yes | Q2 Unclear, Q4 No, Q5 No, Q7 Unclear |
| Li (2025) | Narrative synthesis only | Randomized controlled trial as reported | Unclear | Unclear | Unclear | No | No | Yes | Unclear | Yes | Unclear | Unclear | Unclear | Yes | Yes | Q1 Unclear, Q2 Unclear, Q3 Unclear, Q4 No, Q5 No, Q7 Unclear, Q9 Unclear, Q10 Unclear, Q11 Unclear |
| Lu et al. (2025) | Primary meta-analysis | Randomized controlled trial as reported; randomization process insufficiently... | Yes | Unclear | Yes | No | No | Yes | Unclear | Yes | Yes | Yes | Yes | Yes | Yes | Q2 Unclear, Q4 No, Q5 No, Q7 Unclear |
| Ma et al. (2025a) | Primary meta-analysis | Randomized controlled trial | Yes | Yes | Yes | No | No | Yes | Yes | Yes | Yes | Yes | Yes | Yes | Yes | Q4 No, Q5 No |
| Xu et al. (2025) | Primary meta-analysis | Randomized controlled trial | Yes | Unclear | Yes | No | No | Yes | Unclear | Yes | Yes | Yes | Yes | Yes | Yes | Q2 Unclear, Q4 No, Q5 No, Q7 Unclear |
| Hsia et al. (2024) | Primary meta-analysis | Class-level/cluster randomized controlled trial | Yes | Unclear | Yes | No | No | Yes | Unclear | Yes | Yes | Yes | Yes | Yes | Yes | Q2 Unclear, Q4 No, Q5 No, Q7 Unclear |

Question key: Q1: Was true randomization used for assignment of participants to treatment groups? | Q2: Was allocation to treatment groups concealed? | Q3: Were treatment groups similar at the baseline? | Q4: Were participants blind to treatment assignment? | Q5: Were those delivering the treatment blind to treatment assignment? | Q6: Were treatment groups treated identically other than the intervention of interest? | Q7: Were outcome assessors blind to treatment assignment? | Q8: Were outcomes measured in the same way for treatment groups? | Q9: Were outcomes measured in a reliable way? | Q10: Was follow-up complete and, if not, were differences between groups in terms of their follow-up adequately described and analyzed? | Q11: Were participants analyzed in the groups to which they were randomized? | Q12: Was appropriate statistical analysis used? | Q13: Was the trial design appropriate, and were any deviations from the standard RCT design (individual randomization, parallel groups) accounted for in the conduct and analysis of the trial?

**Table S4b. Quasi-experimental and non-randomized intervention studies (revised JBI quasi-experimental tool).**

| **Study (author–year)** | **Synthesis role** | **Final design** | **Q1** | **Q2** | **Q3** | **Q4** | **Q5** | **Q6** | **Q7** | **Q8** | **Q9** | **Key concerns** |
| --- | --- | --- | --- | --- | --- | --- | --- | --- | --- | --- | --- | --- |
| Huang et al. (2026) | Primary meta-analysis | Quasi-experimental controlled intervention study | Yes | Yes | Yes | Yes | Yes | Yes | Yes | Yes | Yes | No No/Unclear items in the design-specific appraisal. |
| Shen (2026) | Narrative synthesis only | Quasi-experimental / multi-case intervention evaluation | Unclear | No | N/A | N/A | Unclear | Unclear | Unclear | Unclear | No | Q1 Unclear, Q2 No, Q5 Unclear, Q6 Unclear, Q7 Unclear, Q8 Unclear, Q9 No |
| Sun and Lin (2026) | Narrative synthesis only | Quasi-experimental controlled intervention study | Yes | Yes | Yes | Yes | Yes | Yes | Yes | Yes | Yes | No No/Unclear items in the design-specific appraisal. |
| Zhong (2026) | Narrative synthesis only | Quasi-experimental controlled intervention study | Yes | Yes | Yes | Yes | Yes | Yes | Yes | Unclear | Yes | Q8 Unclear |
| Lin et al. (2025) | Primary meta-analysis | Quasi-experimental controlled intervention study | Yes | Yes | Yes | Yes | Yes | Yes | Yes | Yes | Yes | No No/Unclear items in the design-specific appraisal. |
| Zhang et al. (2025) | Narrative synthesis only | Non-randomized implementation / before-after evaluation | Yes | Yes | Yes | N/A | Yes | Yes | Yes | Unclear | Yes | Q8 Unclear |
| Lin (2024) | Narrative synthesis only | Quasi-experimental controlled intervention study | Yes | Yes | Yes | Unclear | Yes | Yes | Unclear | Yes | No | Q4 Unclear, Q7 Unclear, Q9 No |

Question key: Q1: Is it clear in the study what is the 'cause' and what is the 'effect' (i.e., there is no confusion about which variable comes first)? | Q2: Was there a control group? | Q3: Were participants included in any comparisons similar? | Q4: Were the participants included in any comparisons receiving similar treatment/care, other than the exposure or intervention of interest? | Q5: Were there multiple measurements of the outcome, both pre and post the intervention/exposure? | Q6: Were the outcomes of participants included in any comparisons measured in the same way? | Q7: Were outcomes measured in a reliable way? | Q8: Was follow-up complete and, if not, were differences between groups in terms of their follow-up adequately described and analyzed? | Q9: Was appropriate statistical analysis used?

**Table S4c. Analytical cross-sectional/system-validation study (revised JBI analytical cross-sectional tool).**

| **Study (author–year)** | **Synthesis role** | **Final design** | **Q1** | **Q2** | **Q3** | **Q4** | **Q5** | **Q6** | **Q7** | **Q8** | **Key concerns** |
| --- | --- | --- | --- | --- | --- | --- | --- | --- | --- | --- | --- |
| Ma et al. (2025b) | Narrative synthesis only | Analytical cross-sectional / system-validation study | Yes | Yes | Yes | Yes | Yes | Yes | Yes | Yes | No No/Unclear items in the design-specific appraisal. |

Question key: Q1: Were the criteria for inclusion in the sample clearly defined? | Q2: Were the study subjects and the setting described in detail? | Q3: Was the exposure measured in a valid and reliable way? | Q4: Were objective, standard criteria used for measurement of the condition? | Q5: Were confounding factors identified? | Q6: Were strategies to deal with confounding factors stated? | Q7: Were the outcomes measured in a valid and reliable way? | Q8: Was appropriate statistical analysis used?

# Risk-of-Bias Summary (Post-QC Consensus)

| **Design group** | **N studies** | **Yes** | **No** | **Unclear** | **N/A** |
| --- | --- | --- | --- | --- | --- |
| Randomized controlled trials | 10 | 85 | 20 | 25 | 0 |
| Quasi-experimental / non-randomized intervention studies | 7 | 48 | 3 | 9 | 3 |
| Analytical cross-sectional / system-validation study | 1 | 8 | 0 | 0 | 0 |

Post-QC consensus across all 18 retained studies comprised 141 Yes, 34 Unclear, 23 No, and 3 Not applicable item-level judgments (201 items total). No percentage-based overall score or global high/moderate/low quality category was calculated. Common RCT concerns were lack of participant/provider blinding, unclear allocation concealment, and unclear assessor blinding; several quasi-experimental/narrative-only studies additionally had uncertainty in follow-up, outcome reliability, control structure, or statistical analysis. These design and measurement features are also relevant when interpreting unusually large study-level standardized effects, because non-blinded or evaluator-rated assessments and small samples may contribute to upwardly biased or unstable estimates.

# S5 GRADE Certainty-of-Evidence Assessment

GRADE was applied to the primary meta-analytic outcome, post-intervention motor-skill performance. The pooled evidence comprised 11 studies, including nine studies classified as randomized trials and two quasi-experimental studies. The certainty assessment was anchored to the randomized evidence and informed by a randomized-trial-only sensitivity analysis. The final certainty was very low.

**Table S5. GRADE evidence profile for post-intervention motor-skill performance.**

| **GRADE domain** | **Judgment** | **Downgrade** | **Rationale** |
| --- | --- | --- | --- |
| Risk of bias | Serious | -1 | Allocation concealment and outcome-assessor blinding were frequently unclear in randomized studies; participant/provider blinding was not achieved; two quasi-experimental studies also contributed to the pooled estimate. |
| Inconsistency | Very serious | -2 | I² = 94.70%, τ² = 1.50748, and the 95% prediction interval (-1.036 to 4.003) crossed the null and spanned potentially unfavorable to very large positive effects. RCT-only sensitivity remained highly heterogeneous (I² = 95.45%; PI -1.098 to 4.422). |
| Indirectness | Serious | -1 | Nine of 11 meta-analysis studies were conducted in East Asian settings and eight of 11 involved university populations, limiting generalizability to broader school-age and international physical-education contexts. Across the systematic review, 18 of 20 studies (90%) were conducted in mainland China or Taiwan, reinforcing concerns about geographical generalizability. |
| Imprecision | Not serious | 0 | The pooled 95% CI (0.738 to 2.230) remained entirely on the side of benefit. No established minimally important standardized effect threshold was identified for this heterogeneous outcome construct, so uncertainty in exact magnitude was acknowledged without an additional downgrade. |
| Publication bias | No downgrade | 0 | Egger's test p = 0.176 and Begg's test p = 0.052 did not provide compelling evidence of small-study effects, although k = 11 limits the ability to exclude reporting bias. |
| **Overall certainty** | **Very low** | Final | Confidence in the pooled magnitude is very limited. The average effect was positive, but important risk-of-bias, heterogeneity, and generalizability concerns mean that effects may differ substantially across settings. |

Primary pooled estimate: Hedges' g = 1.484 (95% CI [0.738, 2.230]); I² = 94.70%; τ² = 1.50748; 95% prediction interval -1.036 to 4.003. Randomized-trial-only sensitivity (9 studies): g = 1.662 (95% CI [0.768, 2.556]); I² = 95.45%; τ² = 1.77549; 95% prediction interval -1.098 to 4.422. The prediction interval was considered primarily within the inconsistency domain rather than as a substitute for the GRADE imprecision judgment.

# Supplementary Table A1. Detailed study characteristics (18 retained studies).

| Study | Country/Region | Education level | Participants / sample size | Study design | PE or motor-skill context | Sport or motor skill domain | Intervention duration | Comparison condition | Main motor skill outcome | Inclusion type | Notes |
| --- | --- | --- | --- | --- | --- | --- | --- | --- | --- | --- | --- |
| Bouzid et al. (2026) | Tunisia / international | Youth sports education/training | Youth soccer players; n = 23 | Randomized controlled training experiment | Youth soccer tackling skill acquisition; educational sport-skill context | Youth soccer tackling skill acquisition; educational sport-skill context | 6 weeks / 12 training sessions | Verbal feedback only | Tackling technical performance, trunk-leg angle, motivation | Meta-analysis | NR |
| Huang et al. (2026) | Taiwan | University | 80 university students from two yogism classes; n = 80 | Quasi-experimental controlled study | University physical education yogism movement-skill course | University physical education yogism movement-skill course | NR | Conventional PE learning without gamified AI movement assessment and feedback | Yogism movement skills performance | Meta-analysis | NR |
| Lin (2026) | Jilin Province; one higher-education institution | University | Second-year students taking a college public basketball course; n = 216 | Teaching experiment in the 2023 spring semester; students randomly assigned to SCT smart classroom mode or traditional mode; same teacher and controlled class conditions; pre-test showed no significant group differences. | College public basketball; basketball skill test, tactical application, and action standardization | College public basketball; basketball skill test, tactical application, and action standardization | 2023 spring semester; exact week count not reported | Traditional college public basketball teaching | Skill test score; tactical application improvement; action standardization improvement | Meta-analysis | NR |
| Rakha (2026) | Saudi Arabia | University | 56 undergraduate handball beginners; n = 56 | Randomized pre-test/post-test control group design | University handball PE course | University handball PE course | 15-session instructional program | Reciprocal teaching style without ChatGPT-based feedback | Seven fundamental handball skills and composite skill score | Meta-analysis | NR |
| Shen (2026) | Three college smart-sports pilot projects | University | College sports training participants; exact student sample not reported; n = Not reported | Literature analysis, case study, experimental comparison, and 6-month follow-up observation of smart-sports pilot projects | College sports training; examples include swimming, shot put, and basic sports actions | College sports training; examples include swimming, shot put, and basic sports actions | 6 months of tracking observation | Traditional training mode or traditional training stage, but group/sample details are not reported | Action correction efficiency, technical standardization time, physical-fitness standard rate, injury prevention indicators | Systematic review only | NR |
| Sun and Lin (2026) | China | University | Public PE students from a non-PE institute at one university; n = 114 | 14-week quasi-experimental mixed teaching intervention | University PE courses covering basketball, soccer and tennis skill tests | University PE courses covering basketball, soccer and tennis skill tests | 14 weeks; 36 class hours | Traditional classroom teaching mode | Obstacle dribble three-step layup, dribble shot, forehand/backhand reception | Systematic review only | Downgraded to systematic review only |
| Zhong (2026) | One university football elective course; institution not named | University | Two parallel classes of 2024 college football elective students; n = 90 | Experimental comparison of smart sports technology-assisted football teaching versus traditional teaching; 16 weeks; 2 class hours per week; pre-test showed no significant differences. | Football; passing accuracy, shooting accuracy, and slalom dribbling time | Football; passing accuracy, shooting accuracy, and slalom dribbling time | 16 weeks; 2 class hours per week | Traditional football teaching with teacher demonstration and group practice | Passing accuracy, shooting accuracy, slalom dribbling time | Systematic review only | NR |
| Hsia et al. (2025) | Taiwan | University | 88 university students from two yoga courses; n = 88 | Controlled class-based experiment during online PE | Online university PE yoga course | Online university PE yoga course | NR | C-Yoga conventional online yoga learning system with instructor feedback | Yoga skill performance | Meta-analysis | NR |
| Hsu et al. (2025) | Taiwan | University | Students in elementary university badminton classes; n = 138 | Classroom experiment with control and experimental groups | University elementary badminton course | University elementary badminton course | 9 weeks | Traditional verbal instruction without ABCapp | Badminton clear distance and expert manual skill scores | Meta-analysis | NR |
| Li (2025) | Three primary schools in one city | Primary school | Grade 3-5 primary school students aged 8-11; n = 150 | 16-week pre-post intervention experiment; experimental group used intelligent sports equipment; control group received traditional PE | Basic motor skills; speed, strength, flexibility, balance, coordination, running/throwing/jumping examples | Basic motor skills; speed, strength, flexibility, balance, coordination, running/throwing/jumping examples | 16 weeks; 3 sessions per week; 60 minutes per session | Traditional PE teaching | Speed, strength, flexibility, balance, coordination, basic movement skill assessment | Systematic review only | NR |
| Lin et al. (2025) | Taiwan | University | University badminton learners; n = Serve outcome: 89; clear outcome: 96 | Quasi-experimental teaching experiment | Badminton serve and clear skill learning in PE | Badminton serve and clear skill learning in PE | 6 weeks | Mobile video recording/replay and conventional teacher guidance | Badminton serve and clear performance scores | Meta-analysis | NR |
| Lu et al. (2025) | China | High school, university and club programs | Cheerleading learners/athletes aged 14-25; n = 200 | Randomized controlled trial plus deployment/case analyses | Cheerleading technique teaching/training in sports education contexts | Cheerleading technique teaching/training in sports education contexts | 12 weeks | Traditional cheerleading coaching | Final skill score, time to proficiency, retention, biomechanical parameters | Meta-analysis | NR |
| Ma et al. (2025a) | China | University | University Baduanjin course students; n = 60 | 8-week randomized controlled trial with mediation analysis | Online university Baduanjin PE course | Online university Baduanjin PE course | 10-week study; 8-week intervention period | MOOC-only online Baduanjin learning | Baduanjin total performance, movement quality and execution fluency | Meta-analysis | NR |
| Ma et al. (2025b) | China | University | University students in Baduanjin PE; n = Development datasets: n=20 + instructor; later 20 + 35; objective user test n=10 | System development and objective user test | Baduanjin physical education course | Baduanjin physical education course | NR | NR | Movement accuracy assessment and recognition consistency | Systematic review only | NR |
| Xu et al. (2025) | China | Vocational education | 40 vocational-school dance students; n = 40 | Randomized controlled teaching experiment | Dance skill learning; relevant educational motor-skill context | Dance skill learning; relevant educational motor-skill context | NR | Traditional dance teaching | Dance skill performance | Meta-analysis | NR |
| Zhang et al. (2025) | China | University | Students at one university sports platform; n = Not clearly reported | Case study | University physical education platform | University physical education platform | NR | NR | Teaching effectiveness/physical fitness monitoring; no extractable skill outcome | Systematic review only | NR |
| Hsia et al. (2024) | Taiwan | University | 187 university yoga-course students; n = 187 | Controlled class-based experiment | University physical education yoga course | University physical education yoga course | 4-week teaching experiment within a 10-week course | General online learning system | Yoga skills performance | Meta-analysis | NR |
| Lin (2024) | Fuzhou Gezhi Middle School | High school | Senior high school table-tennis option-course students; n = 90 | One-semester experimental comparison of smart table-tennis teaching versus traditional teaching | Table tennis; table-tennis skill-level grading | Table tennis; table-tennis skill-level grading | One semester | Traditional table-tennis teaching without smart PE intervention | Table-tennis skill levels before and after teaching | Systematic review only | NR |

# Note. Studies are identified by author–year citations matching the complete references in the main manuscript; rows are ordered by publication year (newest first) and alphabetically within year.

# Supplementary Table A2. AI technologies, feedback modalities, and study-specific outcomes.

| Study | AI/intelligent technology | AI technology category | Feedback modality | Feedback timing | Automation level | Human teacher involvement | Motor skill domain | Outcome category | Key finding |
| --- | --- | --- | --- | --- | --- | --- | --- | --- | --- |
| Bouzid et al. (2026) | DeepMotion Animate 3D visual feedback | AI 3D motion capture | 3D visual/video | Post-action/during training | Moderate | High | Soccer tackling | Movement quality / technique execution | AI-based 3D visual feedback improved trunk-leg angle and tackling-performance indicators. |
| Huang et al. (2026) | Gamified AI movement assessment and feedback system (G-AI-MAF) | Computer vision / AI assessment | Visual/dashboard/scores/leaderboard | Immediate/real-time | High | Moderate | Yogism movement skills | Movement quality / technique execution | Experimental group showed higher yogism movement skills performance than control group. |
| Lin (2026) | SCT model with smart devices, platform logs, AI feedback and teacher comments | Smart classroom / wearable sensors / AI analytics | Wearables/platform/dashboard | Mixed real-time/process | Moderate | High | Basketball | Movement quality / technique execution | SCT mode improved skill test score and related learning/participation indicators. |
| Rakha (2026) | Free ChatGPT embedded in reciprocal teaching style | Generative AI / chatbot | Text/verbal peer-mediated | During skill phase/immediate | Moderate | High | Handball skills | Sport-specific skill performance | ChatGPT-supported reciprocal teaching improved seven handball skills and composite score. |
| Shen (2026) | AI video, wearable devices, ML, edge computing and AR | Markerless motion capture / AI video analysis | Audio/vibration/AR/wearable | Real-time | High | Moderate | Swimming, shot put and basic actions | Other | Reports AI-assisted action correction and technical-standardization improvements, but group/sample details are incomplete. |
| Sun and Lin (2026) | High-precision motion capture, biomechanical modeling, posture comparison | Virtual simulation / motion capture | VR/3D/sensor feedback | Real-time/immediate | High | Moderate | Basketball, soccer, tennis | Other | Virtual simulation group outperformed control on sport-skill outcomes; exact means/SDs not reported. |
| Zhong (2026) | Action capture, VR tactical simulation and smart monitoring | Smart sports technology / VR / motion analysis | Motion analysis/VR/dashboard | Real-time/process | Moderate | High | Football | Other | Smart sports technology group improved football passing, shooting, and dribbling indicators, but meta data were incomplete in the extraction file. |
| Hsia et al. (2025) | Auto-Yoga system with OpenPose-based assessment | Computer vision / pose estimation | Skeleton visualization/radar chart/ranking | Immediate/after upload | High | Moderate | Yoga skills | Movement quality / technique execution | Auto-Yoga group outperformed C-Yoga group on post-test yoga performance. |
| Hsu et al. (2025) | MediaPipe + key-frame detection model integrated into ABCapp | Computer vision / machine learning | Mobile video/app/visual | Immediate/app-supported | Moderate | High | Badminton clear | Sport-specific skill performance | ABCapp group showed greater clear-distance improvement than control. |
| Li (2025) | Smart basketball, fitness trackers and virtual interactive platforms | Intelligent sports equipment / sensors | Sensor/equipment/platform | Immediate | Moderate | High | Basic motor skills | Fundamental motor skills | Intelligent equipment group showed better basic motor skill development, but meta-ready continuous data were not extracted. |
| Lin et al. (2025) | OpenPose/deep-learning badminton app with WISER model | Deep learning / pose estimation | Mobile app/video/visual feedback | Immediate/automated | High | Moderate | Badminton serve and clear | Sport-specific skill performance | Auto-feedback WISER app produced higher post-test serve and clear scores. |
| Lu et al. (2025) | CNN/RNN, 3D pose estimation and cognitive feedback system | Deep visual recognition / 3D pose estimation | Visual/auditory/haptic | Real-time/immediate | High | Moderate | Cheerleading technique | Other | AI-assisted instruction reduced time to proficiency and improved final skill scores and retention. |
| Ma et al. (2025a) | MediaPipe pose recognition AI real-time feedback system | Computer vision / pose recognition | Visual/dashboard/automated scores | Real-time/immediate | High | Moderate | Baduanjin | Movement quality / technique execution | AI-feedback group improved more in Baduanjin movement specifications, demonstration level, and total score. |
| Ma et al. (2025b) | IMU motion capture formative assessment system | Wearable sensor / IMU | Sensor/dashboard | Real-time | High | Moderate | Baduanjin | Other | IMU-based system supported real-time formative assessment but did not report controlled intervention effect-size data. |
| Xu et al. (2025) | DSTEVF dance skills teaching, evaluation and visual feedback system | AI visual evaluation | Visual/video | Immediate/real-time | High | Moderate | Dance skills | Movement quality / technique execution | DSTEVF-based learning group showed higher post-test dance skill performance. |
| Zhang et al. (2025) | Wearables, edge computing and cloud analysis | Big data / AI platform | Wearable/dashboard/platform | Real-time | Moderate | Moderate | General PE/physical fitness | Other | Case study described AI/big-data PE platform and real-time feedback, without extractable controlled motor-skill data. |
| Hsia et al. (2024) | OpenPose yoga automatic assessment and feedback system | Computer vision / pose estimation | Skeleton visualization/scores/ranking | Immediate/after upload | High | Moderate | Yoga skills | Movement quality / technique execution | AI auto-assessment and feedback group showed higher yoga skills performance. |
| Lin (2024) | Smart table-tennis devices, wristbands, smart resources and platform data | Smart platform / intelligent equipment | Wearables/platform/smart equipment | Mixed/process | Moderate | High | Table tennis | Sport-specific skill performance | Smart-platform group improved table-tennis skill levels relative to control, but effect-size data are not directly usable. |

**Operational classification note.** For this review, AI-based interventions required an AI or intelligent computational function that analysed, interpreted, evaluated, or otherwise transformed learner- or movement-related information for feedback or assessment. Real-time or immediate feedback included feedback delivered during performance or in sufficiently close temporal proximity within the same instructional or practice cycle. Teacher- and peer-mediated delivery were retained when AI-derived information materially informed feedback. The categories in Table A2 describe technological implementation and do not imply technological equivalence. Note. Studies are identified by author–year citations matching the complete references in the main manuscript; rows are ordered by publication year (newest first) and alphabetically within year.

**Outcome-category reconciliation (descriptive versus meta-analytic).** The Outcome category column in Supplementary Table A2 represents the descriptive study-level classification used for the 18-study frequencies in main Table 1 and Section 4.4: movement quality / technique execution (n = 7), sport-specific skill performance (n = 4), Other (n = 6), and fundamental motor skills (n = 1). The selected outcome contributing to the meta-analysis can have a different classification from the broader study-level category. Specifically, Lin (2026) is classified as movement quality / technique execution descriptively, but its selected skill test score is sport-specific skill performance in main Table 2 and Supplementary Table S3; Lu et al. (2025) is classified as Other descriptively, but its selected final cheerleading skill score is sport-specific skill performance; Ma et al. (2025a) is classified as movement quality / technique execution descriptively, but its selected Baduanjin total score is classified as Other in the meta-analysis. For the remaining eight meta-analysed studies, the two categories coincide. The final meta-analytic outcome categories and all numerical effect estimates remain exactly as specified in main Table 2 and Supplementary Table S3. Descriptive study-level frequencies should not be used as meta-analytic subgroup counts.

# PRISMA Nodes Revised

| Node | Count | Breakdown | Notes |
| --- | --- | --- | --- |
| Records identified from databases | 1009 | WOS 374; ERIC 38; PubMed 96; CNKI exported 501 | PubMed uses deduplicated 96 count. |
| Records removed before screening | 182 | Duplicate records 82; CNKI non-journal records 100 | Duplicate records include WOS/ERIC/PubMed 70 and CNKI 12. |
| Records screened | 827 | 438 WOS/ERIC/PubMed + 389 CNKI journal-only | After database-level removals. |
| Records excluded | 725 | WOS/ERIC/PubMed 388; CNKI 337 | Title/abstract screening. |
| Reports sought for retrieval | 102 | WOS/ERIC/PubMed 50; CNKI 52 | WOS/ERIC/PubMed includes Include + Maybe records. |
| Reports not retrieved | 6 | WOS/ERIC/PubMed unavailable 6; CNKI unavailable 0 | Placed in PRISMA reports not retrieved. |
| Reports assessed for eligibility | 96 | WOS/ERIC/PubMed 44 including book/not-journal report; CNKI 52 | 102 reports sought minus 6 not retrieved. |
| Reports excluded with reasons | 78 | WOS/ERIC/PubMed 29; CNKI 47 (including Bi 2025 and Han 2025); book/not journal empirical article 1; retracted article 1 | Full-text/revision eligibility exclusions include two CNKI studies removed after independent eligibility reassessment. |
| Studies included in systematic review | 18 | WOS/ERIC/PubMed contribution 13 after removal of one retracted article and exclusion of Kim_2026; CNKI contribution 5 after excluding Bi_2025 and Han_2025 | Final merged included corpus after reference and two-reviewer revision eligibility verification. |
| Studies included in meta-analysis | 11 | 11 study-level effect sizes | Eleven study-level effects; excluded Bi (2025) and Han (2025) were never part of the primary meta-analytic model. |

# Meta Main Model

| Model | k | Pooled_Hedges_g | SE | CI_Lower | CI_Upper | z_value | p_value | Q | df | Q_p_value | I2_percent | tau2 | tau | Prediction_Interval_Lower | Prediction_Interval_Upper | Tau2_Estimator |
| --- | --- | --- | --- | --- | --- | --- | --- | --- | --- | --- | --- | --- | --- | --- | --- | --- |
| REML random-effects revised after retraction | 11 | 1.483802 | 0.380611 | 0.737818 | 2.229787 | 3.898472 | 9.7e-05 | 188.564955 | 10 | 3.888e-35 | 94.696788 | 1.507481 | 1.227795 | -1.035606 | 4.003211 | REML |

# Retraction Handling Log

| Study_ID | Full citation | Original DOI | Retraction Note DOI | Evidence source | Original inclusion status | Revised inclusion status | Whether excluded from systematic review | Whether excluded from meta-analysis | Changes made to PRISMA | Changes made to meta-analysis | Notes |
| --- | --- | --- | --- | --- | --- | --- | --- | --- | --- | --- | --- |
| Liu_2024 | Liu, F., & Hang, X. (2024). Design of artificial intelligence volleyball teaching intelligent assistant system based on artificial muscle integrated optical equipment. Optical and Quantum Electronics, 56(3), Article 455. https://doi.org/10.1007/s11082-023-06243-0 | 10.1007/s11082-023-06243-0 | 10.1007/s11082-024-07600-3 | Springer original article page; Springer retraction note; Crossref DOI metadata; local PDF first page; Liu_2024_Verification_Report.md | Systematic review and meta-analysis; study-level g=0.907085, CI [0.460551, 1.353619], weight=8.514347% | Excluded after reference verification | Yes | Yes | Retraction stage: SR 22→21; MA 12→11 after removal of the retracted article. Subsequent eligibility verification excluded one qualitative report (SR 21→20), followed by Bi (2025) and Han (2025) (SR 20→18). Final SR n=18, MA k=11. | Removed from study-level main model; REML random-effects model rerun with k=11. | Retracted article; excluded after reference verification. |

**Supplementary Figure S1**. Funnel plot for the revised random-effects meta-analysis.
